# Type I and type V procollagen triple helix uses different subsets of the molecular ensemble for lysine posttranslational modifications in the rER

**DOI:** 10.1016/j.jbc.2021.100453

**Published:** 2021-02-23

**Authors:** Yoshihiro Ishikawa, Yuki Taga, Keith Zientek, Nobuyo Mizuno, Antti M. Salo, Olesya Semenova, Sara F. Tufa, Douglas R. Keene, Paul Holden, Kazunori Mizuno, Douglas B. Gould, Johanna Myllyharju, Hans Peter Bächinger

**Affiliations:** 1 Department of Biochemistry and Molecular Biology, Oregon Health & Science University, Portland, Oregon, USA; 2 Research Department, Shriners Hospital for Children, Portland, Oregon, USA; 3 Department of Ophthalmology, University of California San Francisco, School of Medicine, San Francisco, California, USA; 4 Nippi Research Institute of Biomatrix, Ibaraki, Japan; 5 Oulu Center for Cell-Matrix Research, Biocenter Oulu and Faculty of Biochemistry and Molecular Medicine, University of Oulu, Oulu, Finland; 6 Department of Anatomy, University of California, San Francisco, School of Medicine, San Francisco, California USA

**Keywords:** collagen, posttranslational modifications, lysyl hydroxylase, prolyl hydroxylase, endoplasmic reticulum, molecular chaperone, AAA, amino acid analysis, CRTAP, cartilage-associated protein, CypB, cyclophilin B, ECM, extracellular matrix, EDS, Ehlers–Danlos syndrome, GGHL, glucosylgalactosyl hydroxylysine, LH 1, lysyl hydroxylase 1, P3H3, prolyl 3-hydroxylase 3, PTM, posttranslational modification, rER, rough endoplasmic reticulum, SFM, serum-free media

## Abstract

Collagen is the most abundant protein in humans. It has a characteristic triple-helix structure and is heavily posttranslationally modified. The complex biosynthesis of collagen involves processing by many enzymes and chaperones in the rough endoplasmic reticulum. Lysyl hydroxylase 1 (LH1) is required to hydroxylate lysine for cross-linking and carbohydrate attachment within collagen triple helical sequences. Additionally, a recent study of prolyl 3-hydroxylase 3 (P3H3) demonstrated that this enzyme may be critical for LH1 activity; however, the details surrounding its involvement remain unclear. If P3H3 is an LH1 chaperone that is critical for LH1 activity, P3H3 and LH1 null mice should display a similar deficiency in lysyl hydroxylation. To test this hypothesis, we compared the amount and location of hydroxylysine in the triple helical domains of type V and I collagen from P3H3 null, LH1 null, and wild-type mice. The amount of hydroxylysine in type V collagen was reduced in P3H3 null mice, but surprisingly type V collagen from LH1 null mice contained as much hydroxylysine as type V collagen from wild-type mice. In type I collagen, our results indicate that LH1 plays a global enzymatic role in lysyl hydroxylation. P3H3 is also involved in lysyl hydroxylation, particularly at cross-link formation sites, but is not required for all lysyl hydroxylation sites. In summary, our study suggests that LH1 and P3H3 likely have two distinct mechanisms to recognize different collagen types and to distinguish cross-link formation sites from other sites in type I collagen.

Collagen is not only the most abundant protein but is also one of the most heavily posttranslationally modified proteins in the human body (1, 2). These posttranslational modifications (PTMs) play essential roles in providing biological functions to collagen molecules. Two distinct classifications of PTM exist prior to the formation of functional extracellular matrices (ECMs), occurring in the unfolded state (a single α-chain) in the rough endoplasmic reticulum (rER) and the folded state (triple helical structure) in the Golgi and the ECM space (3, 4). Interestingly, the PTMs in the rER occasionally govern the extent of the PTM in the Golgi and the ECM space. Cross-link formation takes place on side chains of collagen triple helical structure and adds essential biomechanical properties to the collagen fibrils (5, 6). The outcome of cross-link formation in type I collagen depends on the presence or absence of lysyl hydroxylation in both the collagenous and the telopeptide regions (7, 8). Additionally, *O*-glycosylation, which is generated after lysyl hydroxylation, is involved in cross-link formations, and the amount depends on the type of tissue, the rate of triple helix formation, and the presence or absence of ER chaperones (9, 10, 11, 12). Thus, PTMs of unfolded α-chains in the rER are critical for quality control in collagen ultrastructure. In the rER, many enzymes and posttranslational modifiers interact with molecular chaperones *via* either strong or weak affinity interaction to improve their functions (13, 14). In particular, collagen biosynthesis including PTMs is complex and involves many enzymes and chaperones, which are collectively termed the molecular ensemble (15). Because foldases control the rate of triple helix formation and some of the enzymes can only modify unfolded and not triple helical collagen chains, the time that collagen chains remain unfolded in the rER is a critical factor for correct PTMs (16, 17, 18, 19).

Three hydroxylations (proline 3-hydroxylation, proline 4-hydroxylation, and lysine hydroxylation) occur before triple helix formation (3, 15), and the responsible enzymes interact with molecular chaperones *via* a variety of protein–protein interactions (15, 20). When one of these interactions is impaired, as found in genetic disorders, the magnitude of the impact depends on which protein–protein interaction is disrupted. Prolyl 3-hydroxylase 1 (P3H1), cartilage-associated protein (CRTAP), and cyclophilin B (CypB) form a complex with very tight molecular interactions to stabilize each other (21, 22, 23, 24). The lack of one molecule in this complex leads to very phenotypically similar recessive osteogenesis imperfecta (OI) (25, 26). CypB is also identified as a binding partner for lysyl hydroxylase 1 (LH1). CypB is an ER-resident peptidyl-prolyl *cis*-*trans* isomerase and was the first molecule shown to be associated with LH1 (27). CypB was proposed to control lysyl hydroxylation (28), and as such, the absence of CypB or disruption of the interaction with LH1 results in the change of lysyl hydroxylation in the tendon, skin, and bone (28, 29, 30). Lysyl hydroxylase (LH) has three isoforms (LH1, 2, and 3), which hydroxylate specific lysine residues in both the collagenous and telopeptide regions and show collagen sequence preferences (18). LH2 is the only isoform hydroxylating the telopeptide (18), while all three isoforms have been suggested to hydroxylate the triple helical regions (15, 18, 25, 31, 32). Studies of patients with LH1 mutations and of LH3 mutant mice indicate that both LH1 and LH3 could have substrate preferences (*e.g.*, LH1 and LH3 prefer type I/III collagen and type II/IV/V collagen, respectively) (33, 34, 35, 36). However, this indication could not fully explain the diversity of lysyl hydroxylation in tissues of the LH1 null mouse model and type I collagen from different tissues of Ehlers–Danlos Syndrome (EDS)-VIA patients (34, 37). Few analyses have investigated the level of lysyl hydroxylation in purified collagens from mutant or LH knockout models using both qualitative and quantitative measurements. In addition to CypB, two other molecules are likely to be involved in the activity of LH1. SC65, a homolog of CRTAP, is suggested to interact with prolyl 3-hydroxylase 3 (P3H3), which is the other LH1-associated molecule, with relatively strong affinity as indicated by biochemical analyses (38). Additionally, a reduction of lysyl hydroxylation at specific sites was observed in the SC65 null mouse (39). The function of P3H3 is controversial while P3H1 and P3H2 are *bona fide* collagen prolyl 3-hydroxylases. It has been suggested that this protein has no prolyl hydroxylase activity and instead acts as a chaperone for LH1 (39). Collectively, mouse models of LH1 and LH1-associated proteins showed aberrant cross-link formations caused by lysine under hydroxylation at the α1 lysine 87 (K87) and α1 K930 in type I collagen. Therefore, three molecules (CypB, SC65, and P3H3) have been proposed to form a complex with LH1 in the rER (38). However, it is not clear how these molecules interact nor what effect each has on the other.

In this study, we aimed to investigate the precise role of LH1 for PTMs in collagen triple helices and to clarify how tightly P3H3 affects the activity of LH1 in the rER. To achieve these aims, we delved deeply into the levels of overall lysyl hydroxylation and PTM occupancy at individual sites between collagens extracted from LH1 and P3H3 null mice. We analyzed different collagens from different tissues to identify if LH1 is a global or specific enzyme, as described above (33, 34, 35, 36). Moreover, we verified if both P3H3 and LH1 null mice demonstrate similar defects in lysine hydroxylation since P3H3 has recently been suggested to be an essential chaperone required for LH1 activity (39). This direct comparison of different collagens from different tissues from these two null mouse lines provides a significant contribution to understanding the mechanisms for PTM in lysine residues in the rER.

## Results

### Basic characterization of P3H3 null mice

**Figure 1:**
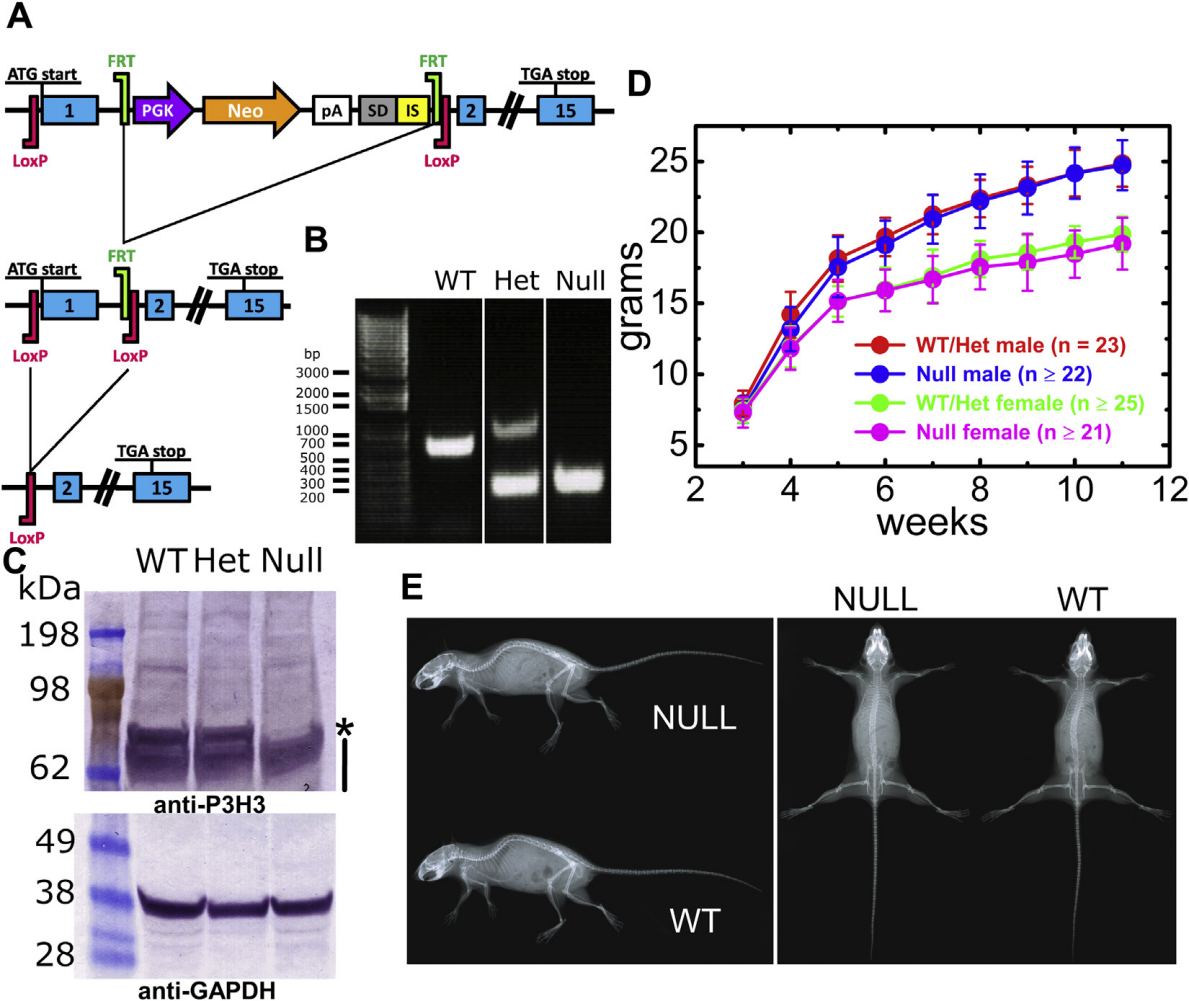
**Generation of P3H3 null mice.***A*, strategy for the generation of the P3H3 null mice. The mouse *Leprel2* gene was eliminated using FRT sites and loxP sites by an FLPe recombinase followed by a Cre recombinase in ES cells, respectively. *B*, PCR genotyping of P3H3 wild-type (WT), heterozygote (Het), and homozygote (Null) mice. Smaller PCR products were generated in Het and Null due to deletion of exon 1. *White spacing* denotes irrelevant lanes that were eliminated from the whole agarose gel image shown in Fig. S1*A*. *C*, total soluble protein of whole mouse kidney from a 3-month-old P3H3 WT, Het, and Null mice was extracted and analyzed by western blotting by using antibodies against P3H3 and GAPDH to confirm equal loading. The *asterisk* and *black line* indicate the P3H3 band and nonspecific bands, respectively. The whole membrane image that was cropped as Figure 1*C* is shown in Fig. S1*B*. *D*, whole body weights of P3H3 WT and Het male (*red*) and female (*green*) and P3H3 Null male (*blue*) and female (*magenta*) were plotted as a function of age. Data are shown as means ± S.D. Growth differences were not observed. *E*, Full body X-rays of 4-month-old P3H3 WT and Null mice were scanned from top and side angles. No skeletal defects were found.

LH1 null mice were generated and characterized previously (37). P3H3 null mice were generated by Ozgene as shown in Figure 1*A* and with more detailed information in the methods section. Table S1 provides the information about tissues from LH1 and P3H3 null mice that were used in this study. Figure 1*B* and Fig. S1*A* display the results of PCR genotyping showing that the P3H3 null allele product is smaller than wild type (WT) due to the deletion of Exon 1. To confirm that the expression of P3H3 protein was abolished, western blotting was performed using a whole kidney lysate, and the protein signal corresponding to the molecular weight of P3H3 (79 kDa) was absent in the P3H3 null lysate (Fig. 1*C* and Fig. S1*B*). Our P3H3 null mice were viable, and we did not observe any apparent growth or skeletal phenotypes by growth curve analysis and X-ray imaging (Fig. 1, *D* and *E* respectively).

### Robust lysyl hydroxylation of skin type V collagen requires P3H3 but not LH1

**Figure 2:**
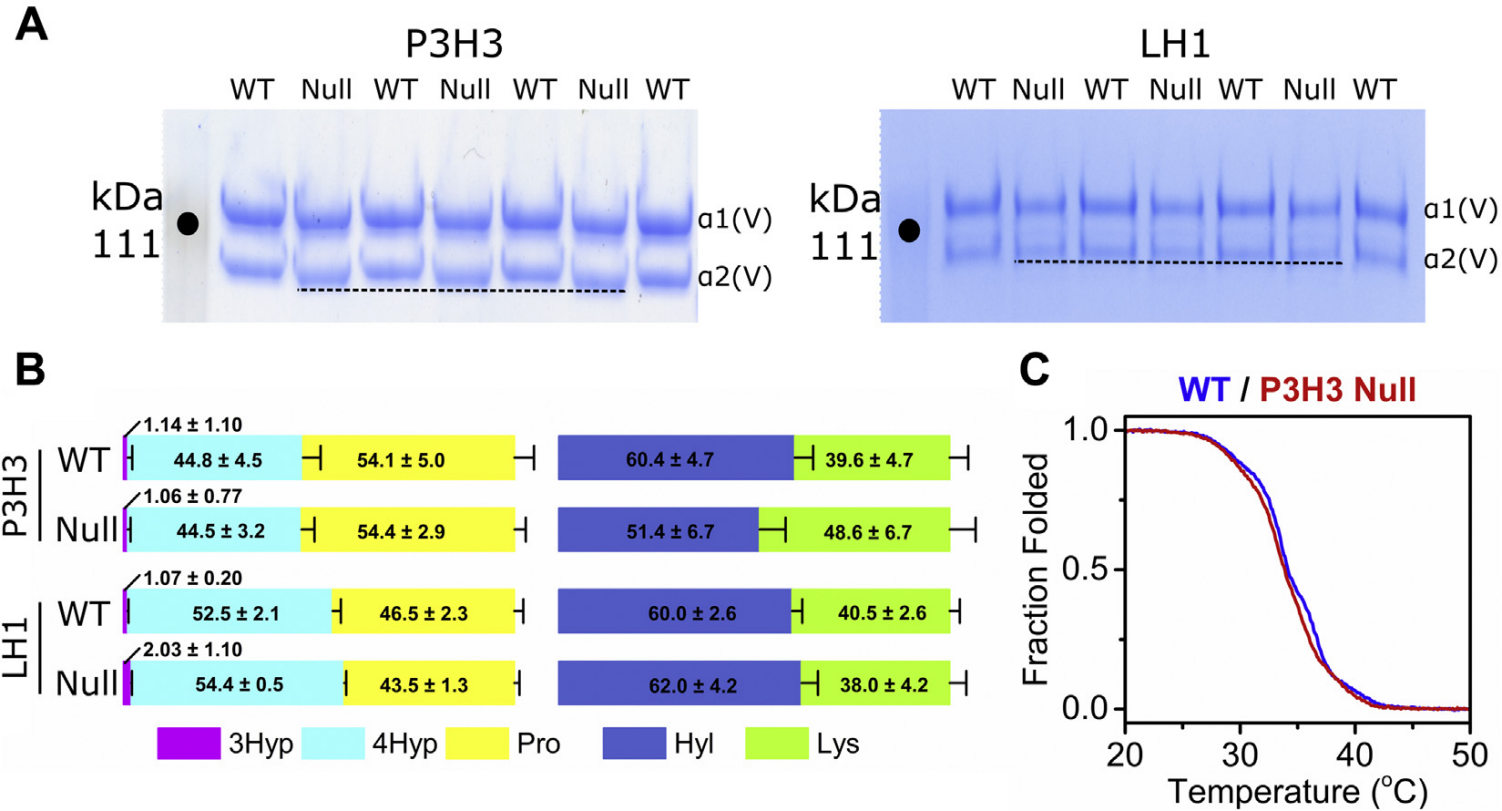
**Characterization of skin type V collagen from P3H3 and LH1 null mice.***A*, magnified image of SDS-PAGE analysis of purified pepsin treated skin type V collagen of P3H3 and LH1 null mice and their WT controls. The purified type V collagen was run on a NuPAGE 3 to 8% Tris-Acetate gel in the presence of a reducing agent and stained with GelCode Blue Stain Reagent. Each sample in the SDS-PAGE gel represents a biological replicate, *i.e.*, an independently prepared collagen sample from the tissue. The *dotted lines* in the gel images indicate the front of the protein bands of the α2 chain of type V collagen. The whole SDS-PAGE images used for Figure 2*A* are shown in Fig. S2. *B*, the ratios of posttranslational modifications in proline (3Hyp + 4Hyp + Pro = 100) and lysine (Lys + Hyl = 100) in skin type V collagen of P3H3 null and LH1 null mice and their WT controls are demonstrated as *bar graphs*, which are generated by values from Table 1. The values of amino acids were obtained using amino acid analysis. The numbers in the graphs indicate the mean ± S.D. of individual amino acids and *p* values obtained by statistical analyses as shown in Table 1. The number of biological replicates is shown in Table 1. [3Hyp(*magenta*); 3-hydroxyproline, 4Hyp (*cyan*); 4-hydroxyprolinen, Pro (*yellow*); unmodified proline, Hyl (*blue*); hydroxylysine, Lys (*green*); unmodified lysine]. *C*, thermal stability of skin type V collagen from P3H3 WT (*blue*) and null (*red*) mice was monitored by CD at 221 nm in 0.05 M acetic acid, and the rate of heating was 10 °C/h. The number of biological replicates for each curve was n = 3 for both WT and P3H3 null. In *A* and *B*, WT samples were separately prepared from P3H3 and LH1 null mouse lines, and skin tissues were collected from 2- to 5-month-old mice.

To investigate the individual and/or cooperative roles of LH1 and P3H3 on lysyl hydroxylation in the collagen triple helix, we performed qualitative and quantitative analyses of type V collagen, since type V collagen is heavily lysyl hydroxylated and *O*-glycosylated and can be isolated to high purity from the skin (40). We purified type V collagen from the skin of P3H3 and LH1 null mice by pepsin treatment followed by sodium chloride precipitation and confirmed the purity by SDS-PAGE analysis (Fig. S2). Interestingly, the α2 chain of type V collagen from P3H3 null mice appeared to migrate slightly faster compared with WT, while the α2 chain of type V collagen from LH1 null mice did not show a clear difference in gel migration (Fig. 2*A*). A difference in collagen migration in SDS-PAGE gels is one of the classic indications of altered levels of PTMs, particularly in the case of OI (41). To explain these observations, we characterized the level of PTMs in both P3H3 null and LH1 null type V collagens by amino acid analysis (AAA), which is used to quantify the total number of PTMs in collagens. The ratios of prolyl hydroxylations look slightly different between WT mice from the P3H3 and LH1 mouse models (Fig. 2*B*). Both mouse lines are in a C57BL/6 background, but slight differences between their backgrounds exist as the mouse lines originated from different laboratories (see Experimental procedures). However, the calculated *p* values are higher than or close to 0.05. Also, the ratios of lysine and hydroxylysine do not show any statistically significant difference between the WT mice (Table S2). Therefore, we decided that it was acceptable to compare the two mouse models. Moreover, corresponding strain specific WT controls were used throughout the study. Surprisingly, P3H3 null mice, but not LH1 null mice, had significantly reduced levels of lysyl hydroxylation relative to their WT controls in type V collagen isolated from the skin, while neither P3H3 nor LH1 null mice had changes in proline hydroxylations (prolyl 3- and 4-hydroxylation) (Fig. 2*B* and Table 1). Type V collagen contains large amounts of *O*-glycosylation on hydroxylysine (40). However, the occupancy of *O*-glycosylation was not changed between WT and P3H3 null mice, even though total hydroxylysine was decreased in P3H3 null mice (Fig. S3). To assess the potential effect of reduced lysyl hydroxylation on type V collagen in P3H3 null mice, we measured the thermal stability of type V collagen by circular dichroism (CD) spectroscopy (42, 43). In the CD melting curve of type V collagen from P3H3 null mice, we found only one thermal transition although we saw two transitions two in WT (Fig. 2*C*); however, this difference is not statistically significant (Fig. S4).

**Table 1:** Comparison of overall proline and lysine posttranslational modifications in type I collagen between tissues and type V collagen in the skin from P3H3 and LH1 null mice relative to their wild-type controls

				3Hyp (%)	*p* value	4Hyp (%)	*p* value	Pro (%)	*p* value	Hyl (%)	*p* value	Lys (%)	*p* value
Col5	skin	P3H3	WT (5)	1.14 ± 1.10	0.84942	44.8± 4.5	0.89295	54.1 ± 5.0	0.85208	60.4 ± 4.7	7.65284E-5	39.6 ± 4.7	7.65287E-5
			Null (17)	1.06 ± 0.77		44.5± 3.2		54.4 ± 2.9		51.4 ± 6.7		48.6 ± 6.7	
		LH1	WT (3)	1.07 ± 0.20	0.1898	52.5 ± 2.1	0.05353	46.5 ± 2.3	0.03944	60.0 ± 2.6	0.41188	40.5 ± 2.6	0.41188
			Null (6)	2.03 ± 1.10		54.4 ± 0.5		43.5 ± 1.3		62.0 ± 4.2		38.0 ± 4.2	
Col1	skin	P3H3	WT (4)	0.17 ± 0.17	0.09497	47.1 ± 3.3	0.58368	52.7 ± 3.5	0.69906	16.9 ± 4.4	1.16563E-5	83.1 ± 2.6	1.16563E-5
			Null (8)	0.37 ± 0.17		46.2 ± 2.5		53.4 ± 2.7		4.0 ± 1.3		96.0 ± 1.3	
		LH1	WT (4)	0.57 ± 0.27	0.32036	51.1 ± 1.1	0.87037	48.3 ± 1.3	0.75609	18.2 ± 2.4	3.02903E-9	81.7 ± 2.4	2.54156E-9
			Null (8)	0.46 ± 0.09		51.0 ± 0.8		48.5 ± 0.8		1.9 ± 0.4		98.1 ± 0.4	
	tendon	P3H3	WT (6)	0.95 ± 0.57	0.66313	44.5 ± 2.7	0.64615	54.6 ± 3.4	0.6146	19.7 ± 2.6	4.01484E-7	80.3 ± 2.5	2.48886E-7
			Null (8)	1.06 ± 0.42		45.1 ± 2.2		53.8 ± 2.6		9.8 ± 1.0		90.2 ± 0.9	
		LH1	WT (6)	1.17 ± 0.26	0.67157	47.2 ± 0.9	0.45461	51.7 ± 1.1	0.49025	18.1 ± 0.6	2.98116E-20	81.9 ± 0.6	2.98116E-20
			Null (12)	1.24 ± 0.39		46.7 ± 1.4		52.1 ± 1.2		1.6 ± 0.5		98.4 ± 0.5	
	bone	P3H3	WT (5)	1.46 ± 0.84	0.79823	48.1 ± 1.7	0.59762	50.5 ± 2.1	0.50736	22.0 ± 3.3	8.81801E-4	78.0 ± 3.3	8.81801E-4
			Null (5)	1.28 ± 1.26		47.2 ± 2.9		51.7 ± 3.5		11.8 ± 3.0		88.2 ± 3.0	
		LH1	WT (5)	0.85 ± 0.53	0.43429	48.0 ± 1.2	0.00101	51.1 ± 1.5	0.00811	18.7 ± 2.8	3.73041E-5	81.3 ± 2.6	2.35247E-5
			Null (4)	1.24 ± 0.87		43.1 ± 1.6		55.6 ± 2.2		4.6 ± 1.1		95.5 ± 1.3	

3Hyp, 3-hydroxyproline; 4Hyp, 4-hydroxyproline; Hyl, hydroxylysine; Lys, unmodified lysine; Pro, unmodified proline.

Values are given as means ± S.D. The number in the bracket next to the genotypes indicates biological replicates.

3Hyp + 4Hyp + Pro = 100% and Lys + Hyl = 100%. Values of amino acids were obtained using amino acid analysis.

**Figure 3:**
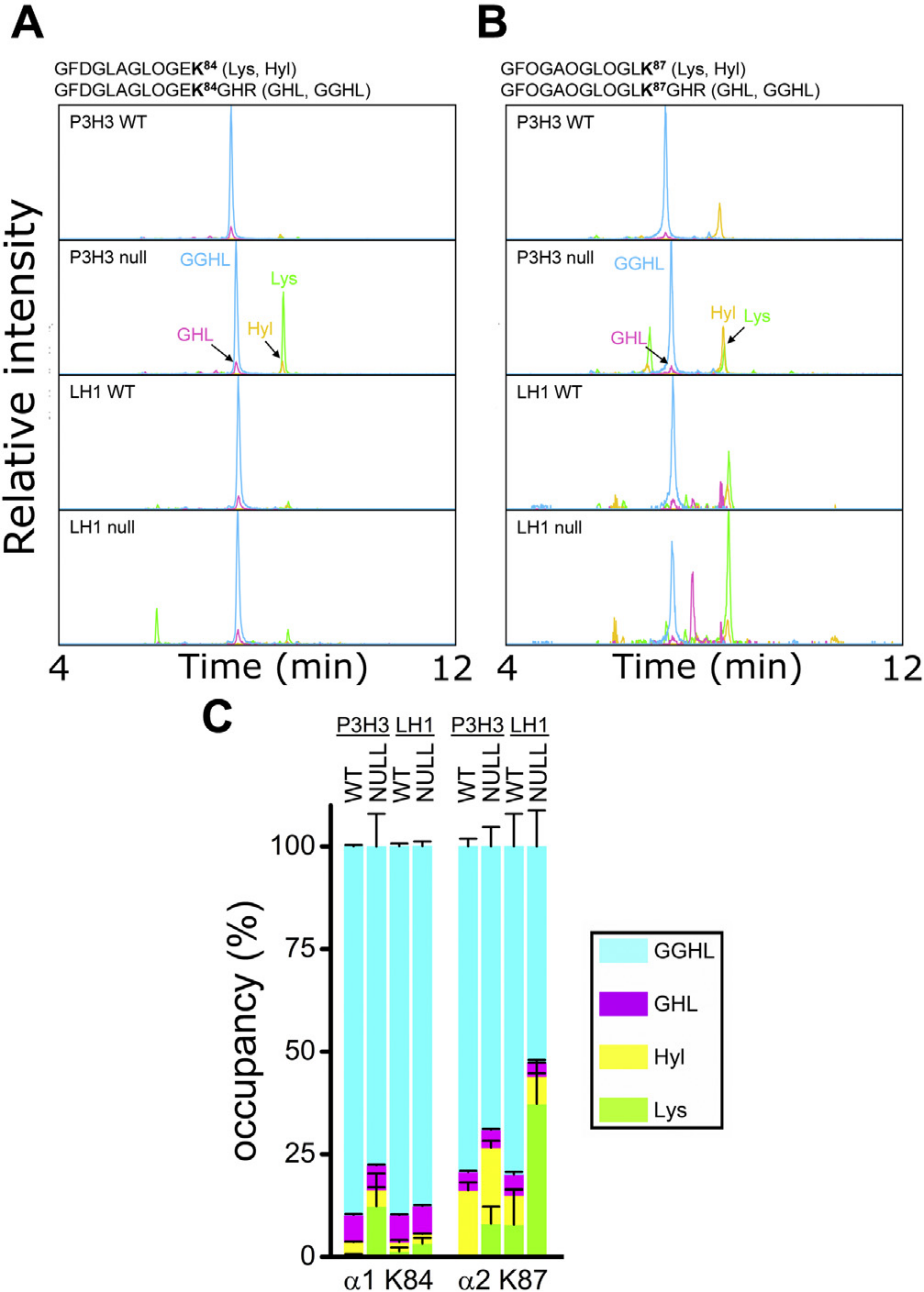
**Characterization of lysine posttranslational modifications at individual sites of skin type V collagen from P3H3 and LH1 null mice.***A*, representative extracted ion chromatograms of α1(V) K84-containing tryptic peptides (*m/z* 588.7911 ± 0.02 (*z* = 2) for Lys, *m/z* 596.7886 ± 0.02 (*z* = 2) for Hyl, *m/z* 568.9372 ± 0.02 (*z* = 3) for GHL, and *m/z* 622.9548 ± 0.02 (*z* = 3) for GGHL). *B*, representative extracted ion chromatograms of α2(V) K87-containing tryptic peptides (*m/z* 579.8040 ± 0.02 (*z* = 2) for Lys, *m/z* 587.8015 ± 0.02 (*z* = 2) for Hyl, *m/z* 562.9458 ± 0.02 (*z* = 3) for GHL, and *m/z* 616.9634 ± 0.02 (*z* = 3) for GGHL). *C*, *bar graphs* represent the occupancy of lysine modifications in individual lysyl hydroxylation sites of skin type V collagen of P3H3 null and LH1 null mice and their WT controls. Individual values [GGHL (*cyan*); glucosylgalactosyl hydroxylysine, GHL (*magenta*); galactosyl hydroxylysine, Hyl (*yellow*); unmodified hydroxylysine, Lys (*green*); unmodified lysine] correspond to Table 2. Values of amino acids were obtained using mass spectrometry, and biological replicates were as shown in Table 2. α1, α2, and K + numbers indicate the α1 and α2 chain of type V collagen and residue number from the first residue of the triple helical domain, respectively.

Next, we determined the occupancy of PTMs at individual lysyl hydroxylation sites in type V collagen from WT, P3H3, and LH1 null mice by liquid chromatography–mass spectrometry (LC-MS) after trypsin digestion. Since lysine residues at the Yaa position of collagenous Gly-Xaa-Yaa triplets are extensively glycosylated in type V collagen (40), site-specific characterization of lysine modifications was difficult due to missed cleavage at hydroxylysine glycosides by trypsin (44). We were able to analyze two glycosylation sites, α1(V) K84 and α2(V) K87, that are involved in cross-link formation (45). Although glycosylated peptides containing these sites were not identified by database search of obtained MS/MS spectra, we manually confirmed the sequence and modification (Fig. S5). As shown by the representative extracted ion chromatograms of each modification form (Fig. 3, *A* and *B*), the level of glucosylgalactosyl hydroxylysine (GGHL) was decreased at both sites in P3H3 null mice, although the decrease at α1(V) K84 did not quite reach statistical significance (Fig. 3*C* and Table 2). The magnitude of reduction corresponds to the increase of unmodified lysines in the absence of P3H3. In LH1 null mice, while there was a marginal change at α1(V) K84, α2(V) K87 was markedly affected (Fig. 3*C* and Table 2). A potential explanation is that the α2-chain of type V collagen is classified as an A-clade chain, which includes both the α1-and α2-chain of type I collagen, whereas the α1-chain of type V collagen belongs to B-clade (46, 47). LH1 seems to hydroxylate the α2(V) K87 preferentially. Nevertheless, the ratio of two α1-chains and one α2-chain in type V collagen could hide the effect caused by impaired LH1 activity and not show any distinct difference in type V collagen between WT and LH1 null as observed in Figure 2*B*. In summary, P3H3 is required for robust lysyl hydroxylation of type V collagen in the skin, whereas LH1 is mostly dispensable for overall lysyl hydroxylation of skin type V collagen but has a specific role in the hydroxylation of specific site(s), at least α2(V) K87.

**Table 2:** Comparison of lysine posttranslational modifications at individual sites of skin type V collagen from P3H3 and LH1 null mice relative to their wild-type controls

			Lys (%)	*p* value	Hyl (%)	*p* value	GHL (%)	*p* value	GGHL (%)	*p* value
α1 K84	P3H3	WT (3)	0.63 ± 0.04	0.06323	2.97 ± 0.09	0.05873	6.61 ± 0.37	0.23762	89.8 ± 0.36	0.05822
		Null (3)	12.3 ± 7.94		3.95 ± 0.65		6.01 ± 0.56		77.6 ± 8.01	
	LH1	WT (4)	1.33 ± 0.92	0.05917	2.28 ± 0.48	0.92243	6.56 ± 0.17	0.06623	89.9 ± 0.66	0.01655
		Null (4)	3.25 ± 1.38		2.30 ± 0.12		6.88 ± 0.21		87.6 ± 1.18	
α2 K87	P3H3	WT (3)	0	0.02794	16.2 ± 1.95	0.17434	4.55 ± 0.22	0.26084	79.3 ± 1.88	0.0248
		Null (3)	8.07 ± 4.14		18.6 ± 1.59		4.55 ± 0.17		69.0 ± 4.71	
	LH1	WT (4)	7.87 ± 8.46	0.00938	7.13 ± 1.51	0.61402	5.07 ± 0.65	0.01839	79.9 ± 7.92	0.00827
		Null (4)	37.3 ± 1.00		6.68 ± 0.74		3.33 ± 0.67		52.7 ± 8.79	

GGHL, glucosylgalactosyl hydroxylysine; GHL, galactosyl hydroxylysine; Hyl, unmodified hydroxylysine; Lys, unmodified lysine.

Values are given as means ± S.D. The number in the bracket next to the genotypes indicates biological replicates.

Lys + Hyl + GHL + GGHL = 100%. Values of modified and unmodified hydroxylysine and unmodified lysine were obtained using mass spectrometry.

### Precise lysyl hydroxylation of type I collagen requires cooperation between P3H3 and LH1

**Figure 4:**
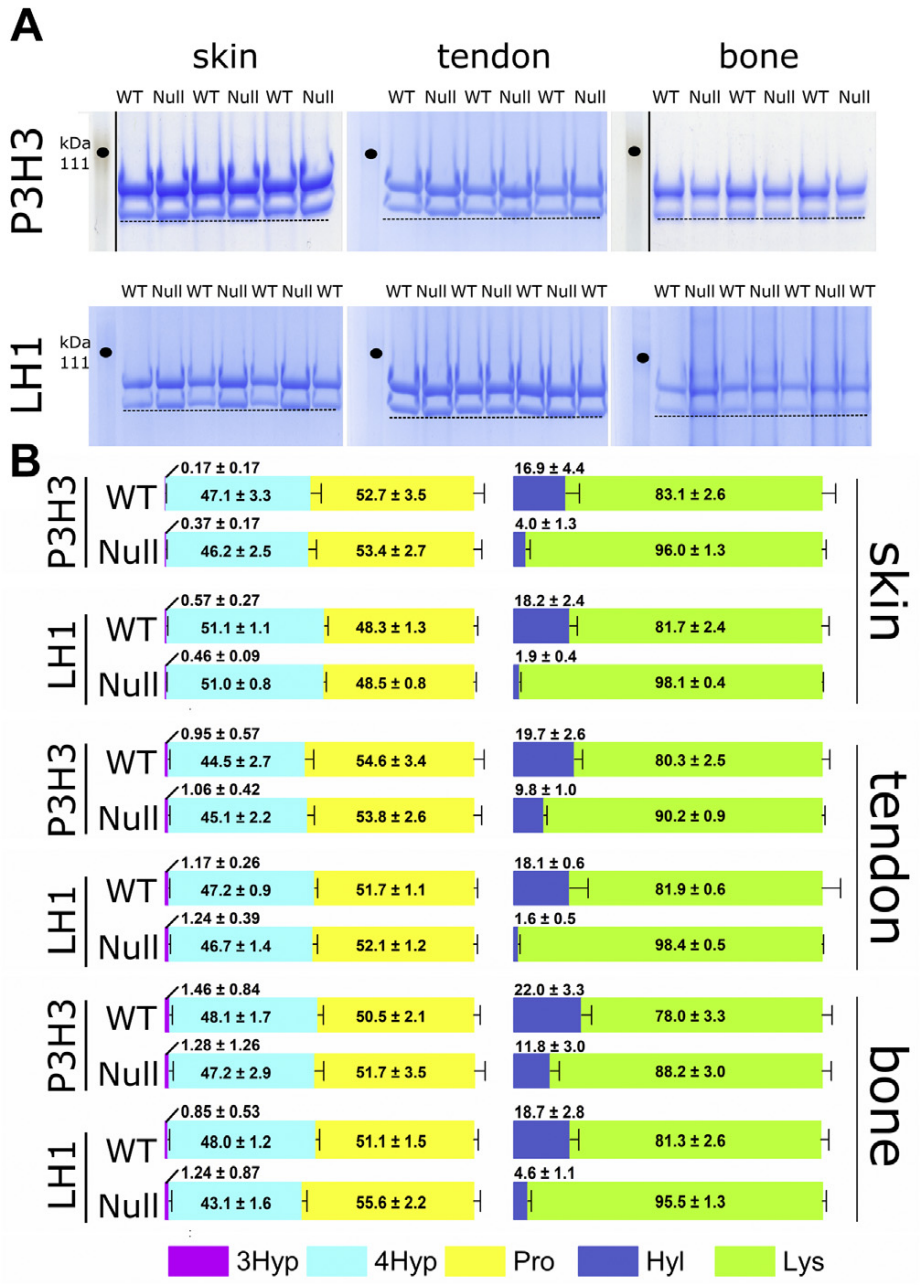
**Biochemical characterization of type I collagen from P3H3 and LH1 null mice.***A*, magnified image of SDS-PAGE analysis of purified pepsin-treated skin type I collagen of P3H3 and LH1 null mice and their WT controls. The purified type I collagen was run on a NuPAGE 3 to 8% Tris-Acetate gel in the presence of a reducing agent and stained with GelCode Blue Stain Reagent. Each sample in the SDS-PAGE gel represents a biological replicate, *i.e.*, an independently prepared collagen sample from the tissue. The *dotted lines* in the gel images indicate the front of the protein band of the α2 chain of type I collagen. The *black vertical lines* in the panels of P3H3 skin and bone denote irrelevant lanes that were eliminated from the original image. The whole SDS-PAGE images used for Figure 4*A* are shown in Fig. S6. *B*, the bar graphs demonstrate the summary of overall proline and lysine posttranslational modifications in type I collagen between genotypes and tissues. The ratio of posttranslational modifications in proline (3Hyp + 4Hyp + Pro = 100) and lysine (Lys + Hyl = 100) in the skin, tendon, and bone type I collagen of P3H3 null and LH1 null mice and their WT controls is demonstrated as bar graphs generated by values from Table 1. The values of amino acids were obtained using amino acid analysis. The numbers in the graphs indicate the mean ± S.D. of individual amino acids and *p* values obtained by statistical analyses as shown in Table 1. The number of biological replicates is shown in Table 1. [3Hyp (*magenta*); 3-hydroxyproline, 4Hyp (*cyan*); 4-hydroxyproline, Pro (*yellow*); unmodified proline, Hyl (*blue*); hydroxylysine, Lys (*green*); unmodified lysine].

As we found distinct differences for collagen type V preference in the lysyl hydroxylase activity of LH1 and P3H3 null mice, we next performed a detailed characterization of PTMs in type I collagen from LH1 null mice and P3H3 null mice. Since previous studies suggested that tissue-specific differences in PTMs exist in type I collagen (48), we examined three different tissues (tendon, skin, and bone) from each mouse model. Using the same approach as for type V collagen, type I collagen was purified from the tissues by pepsin treatment followed by sodium chloride precipitation (Fig. S6) and analyzed qualitatively and quantitatively by SDS-PAGE and AAA, respectively. The tissues from the P3H3 and LH1 mouse models were harvested at different ages, as described in Table S1. However, the levels of prolyl hydroxylations and lysyl hydroxylation were identical between WTs from the P3H3 and LH1 mouse strains (Table S2). Our results thus correspond to a previous report suggesting that the prolyl hydroxylations and lysyl hydroxylation in type I collagen become stable after the early stage (<2 months of age) of tissue development (49). This enabled us to compare type I collagens from the two mouse lines directly. Type I collagen chains from both P3H3 null and LH1 null skin and tendon migrate a little faster, whereas there are no apparent differences in the gel migration of type I collagen from bone between the WT and nulls (Fig. 4*A*). AAA was carried out to quantify the total number of PTMs in the purified type I collagen. Neither P3H3 nor LH1 null mice had consistent changes in proline hydroxylations (prolyl 3- and 4-hydroxylation) (Fig. 4*B* and Table 1). However, both mouse models had interesting changes in lysyl hydroxylation (Fig. 4*B* and Table 1). LH1 deficiency significantly decreased the amounts of hydroxylysine in the tendon, skin, and bone, although bone was affected to a slightly lesser extent. On the other hand, P3H3 deficiency had a much smaller effect on lysyl hydroxylation than LH1, the largest reducing effect being seen in the skin.

**Figure 5:**
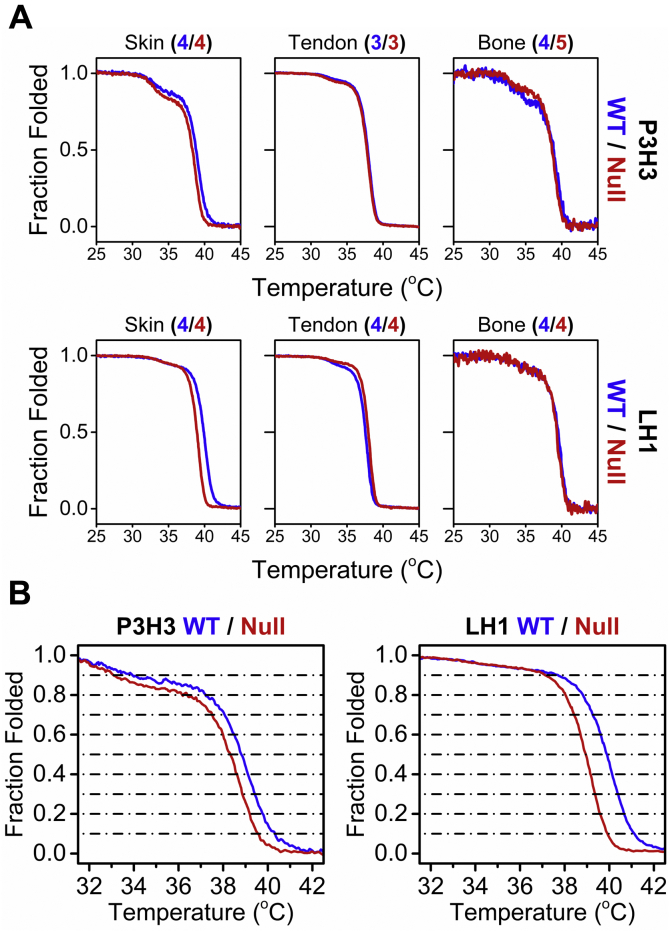
**Comparison of type I collagen melting temperature in different tissues from P3H3 null and LH1 null mice.***A*, the thermal stability of type I collagen from the skin, tendon, and bone from WT (*blue*) and null (*red*) mice was monitored by CD at 221 nm in 0.05 M acetic acid at 10 °C/h heating rate. The *upper* and *lower panels* display P3H3 and LH1 mouse lines, respectively. The number of biological replicates (independently prepared tissue samples) used to generate each curve are shown in the *bracket* next to tissue information with colors corresponding to the genotypes as described above. *B*, magnification of the curves in (*A*) demonstrating the thermal transition between 31 and 43 °C of skin type I collagen from P3H3 and LH1 null mice and their WT controls.

Next, we determined the occupancy of the *O*-glycosylation of hydroxylysine in the skin and tendon by LC-MS. The calculated ratio of GGHL, galactosyl hydroxylysine (GHL), and unmodified hydroxylysine showed a difference with statistical significance between the genotypes in both tissues (Figs. S7 and S8). However, in both mouse models and tissues, the magnitude of change in total hydroxylysine looked more critical than that in occupancy of the *O*-glycosylation of hydroxylysine. Therefore, the difference of *O*-glycosylation seems to provide only a marginal impact to the overall structure of type I collagen in both P3H3 and LH1 null mice (Figs. S7 and S8). To evaluate the impact of the smaller amount of lysyl hydroxylation on the collagen ultrastructure, we measured the thermal stability of type I collagen using CD. Figure 5*A* displays the CD melting curves in three different tissues of P3H3 and LH1 null mice. Clear differences were detected in skin type I collagen of both P3H3 and LH1 null mice. In contrast to skin type V collagen, these differences showed statistical significance, and the reduction of lysyl hydroxylation caused by the absence of P3H3 and LH1 destabilized the collagen triple helix in the skin (Fig. 5*B* and Table 3). This suggests that skin is the most affected tissue in both P3H3 null and LH1 null mice. Taken together, P3H3 has a role in skin type I collagen lysyl hydroxylation, but the level of lysyl hydroxylation is more drastically decreased in LH1 null mice compared with P3H3 null mice.

**Table 3:** The melting temperature of each fraction folded in skin type I collagen from P3H3 and LH1 null mice relative to their wild-type controls

Fraction folded	P3H3			LH1		
	Temperature (°C)		*p* value	Temperature (°C)		*p* value
	Null (4)	WT (4)		Null (3)	WT (3)	
0.9	33.0 ± 0.41	34.5 ± 1.44	0.09	37.0 ± 0.31	37.4 ± 0.96	0.45
0.8	36.1 ± 0.79	37.2 ± 0.39	0.059	38.0 ± 0.08	38.8 ± 0.16	1.21E-04
0.7	37.5 ± 0.24	38.1 ± 0.27	1.68E-03	38.4 ± 0.06	39.3 ± 0.04	2.92E-07
0.6	38.0 ± 0.09	38.5 ± 0.19	2.08E-03	38.7 ± 0.04	39.6 ± 0.07	5.65E-07
0.5	38.3 ± 0.13	38.8 ± 0.11	7.30E-04	38.9 ± 0.05	39.9 ± 0.04	1.22E-07
0.4	38.6 ± 0.08	39.2 ± 0.12	3.35E-04	39.2 ± 0.05	40.1 ± 0.01	1.76E-08
0.3	38.9 ± 0.07	39.5 ± 0.15	5.45E-04	39.3 ± 0.05	40.4 ± 0.05	1.59E-07
0.2	39.2 ± 0.07	39.9 ± 0.22	8.99E-04	39.6 ± 0.05	40.7 ± 0.04	5.68E-08
0.1	39.6 ± 0.19	40.5 ± 0.28	1.97E-03	39.9 ± 0.07	41.1 ± 0.06	2.09E-07

Values are given as means ± S.D. The number in the bracket next to the genotypes indicates biological replicates.

### Global effect and site preference effect on lysyl hydroxylation of type I collagen in P3H3 null and LH1 null tissues

**Figure 6:**
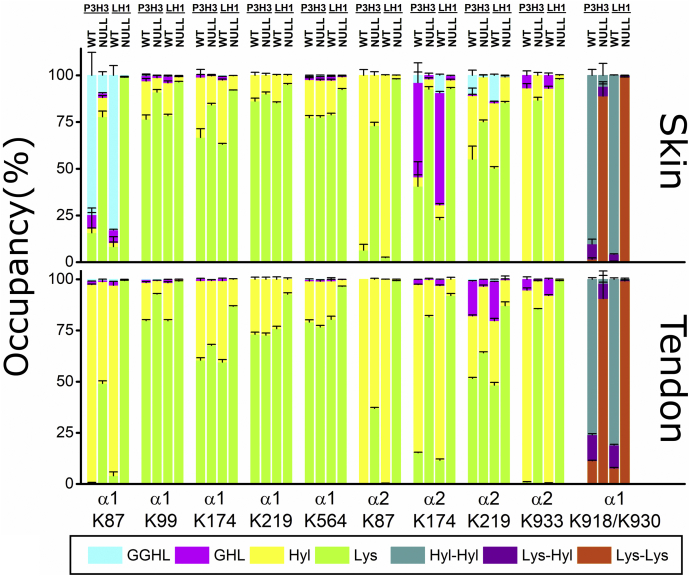
**Characterization of lysine posttranslational modifications at individual sites of skin and tendon type I collagen from P3H3 and LH1 null mice.***Bar graphs* represent the occupancy of lysine modifications in individual lysyl hydroxylation sites of the skin and tendon type I collagen of P3H3 null and LH1 null mice and their WT controls. The lysine modification sites α1 K918 and K930 were included in the same peptide GDKGETGEQGDRGIKGER. Individual values [GGHL (*cyan*); glucosylgalactosyl hydroxylysine, GHL (*magenta*); galactosyl hydroxylysine, Hyl (*yellow*); unmodified hydroxylysine, Lys (*green*); unmodified lysine, Hyl-Hyl (*dark cyan*); unmodified hydroxylysine and unmodified hydroxylysine, Lys-Hyl (*purple*); unmodified lysine and unmodified hydroxylysine, Lys-Lys (*orange*); unmodified lysine and unmodified lysine] correspond to Tables 4 and 5 for the skin and tendon, respectively. Values of modified and unmodified hydroxylysine and unmodified lysine were obtained using mass spectrometry, and the number of biological replicates was n = 4 for all tissues and genotypes. α1, α2, and K + numbers indicate the α1 and α2 chain of type I collagen and residue number from the first residue of the triple helical domain, respectively.

**Figure 7:**
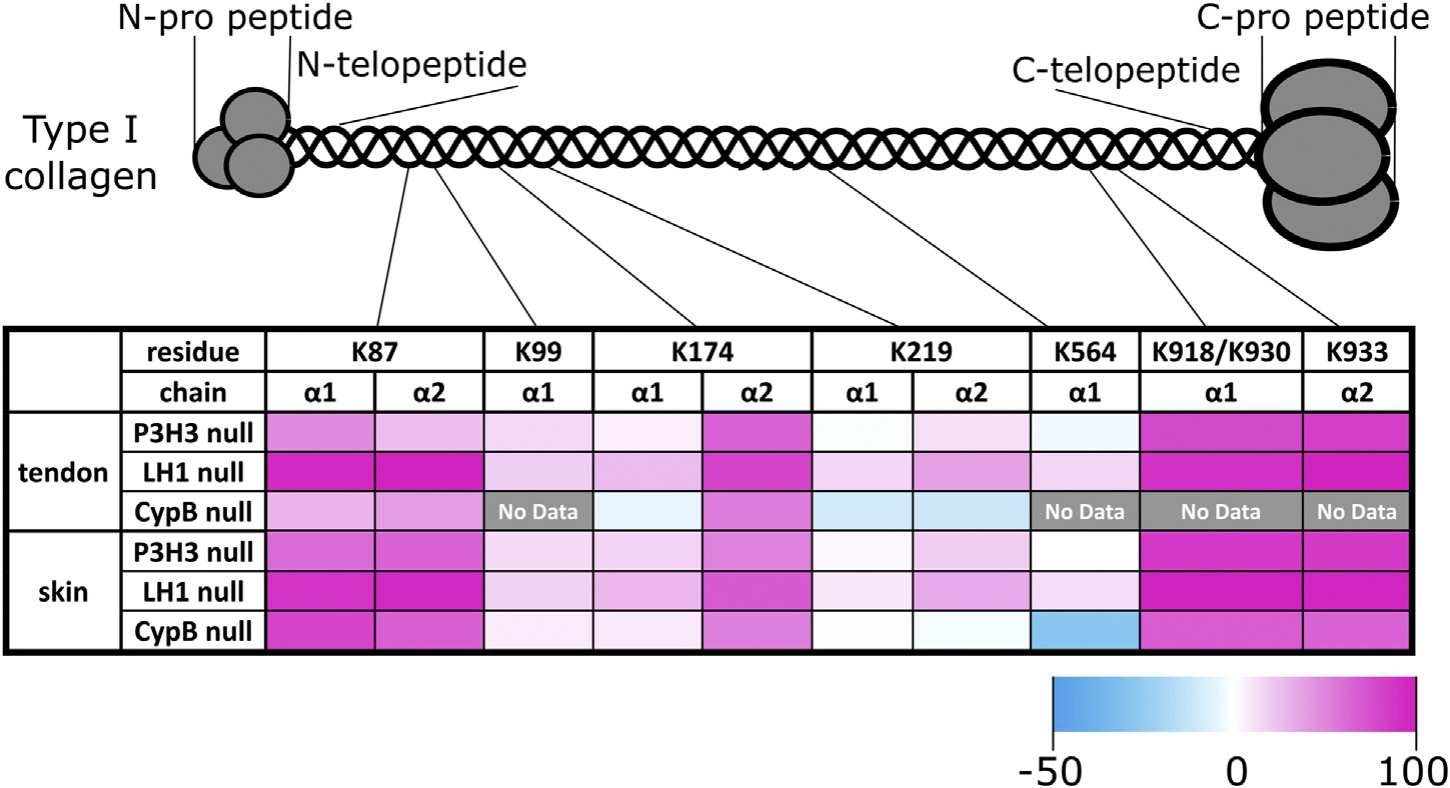
**Magnitudes of change in unmodified lysine residues at individual lysyl hydroxylation sites of type I collagen between P3H3, LH1, and CypB null mice compared with WT.** The schematic diagram and heat map show the magnitudes of change of unmodified lysine residues at individual lysyl hydroxylation sites. The change was calculated by [the value of unmodified lysine (%) in null mice] − [the value of unmodified lysine (%) in WT mice]. The values of the skin and tendon in Tables 4 and 5 were used to calculate the changes in P3H3 null and LH1 null mice. The values of unmodified lysine for CypB null mice tendon and skin were obtained from references (28, 29), respectively.

The α1 and α2 chains of type I collagen contain multiple lysyl hydroxylation sites in their triple helical sequences (28, 29, 48). A previous report of P3H3 null mice (39) only analyzed lysyl hydroxylation of the site K87 in both the α1 and α2 chains. These residues at the amino terminus of the triple helical domain are essential for collagen cross-link formation (39). We determined the occupancy of PTMs at individual lysyl hydroxylation sites between the WT, P3H3, and LH1 null mice in the tendon and skin (Fig. 6, Tables 4 and 5). In LH1 null tissues, the levels of lysyl hydroxylation and subsequent *O*-glycosylation were significantly decreased at all lysyl hydroxylation sites in both the tendon and skin. In P3H3 null tissues, we confirmed the reduction of lysine modifications in K87 in both the α1 and α2 chains of type I collagen as previously reported (39), and a considerable decrease was also found at α1 K930 and α2 K933, which are near the carboxy terminus of the triple helical domain involved in cross-link formation. The sites α1 K99, α1 K174, α1 K918, α2 K174, and α2 K219 also showed an apparent reduction. However, there was no notable decrease in the level of lysyl hydroxylation at the sites in the middle of the triple helix of the α1 chain (α1 K219 and α1 K564). CypB has been proposed to control lysyl hydroxylation, and the occupancy of PTMs at individual lysyl hydroxylation sites in the tendon and skin has been well characterized in CypB null mice (28, 29), as we described for P3H3 and LH1 null mice in Figure 6. To compare the data from the three different null mouse models (P3H3, LH1, and CypB), we focused on the magnitudes of change of unmodified lysine residues in individual lysyl hydroxylation sites between these null mice compared with their WT counterparts (Fig. 7). These observations imply that both P3H3 and CypB play important roles for the function of LH1, and a lack of even one of the components clearly attenuates hydroxylysine formation in the cross-linking sites. Conversely, other lysyl hydroxylation sites demonstrate remarkably diverse effects between the P3H3 null, LH1 null, and CypB null mouse tissues. Overall, each null mouse model has specific pattern changes. Modified lysine residues were hardly found in LH1 null mice, whereas P3H3 null mice showed normal or slightly increased amounts of unmodified lysine residues. In contrast, CypB null mice showed normal or decreased unmodified lysine residues despite a similar increase in unmodified lysine residues at the cross-link formation sites as in P3H3 and LH1 null mice. In summary, LH1 is likely to play a global role for lysyl hydroxylation at all sites in the triple helical domain of type I collagen, whereas the roles of P3H3 and CypB are restricted to specific lysyl hydroxylation sites.

**Table 4:** Comparison of lysine posttranslational modifications at individual sites of skin type I collagen from P3H3 and LH1 null mice relative to their wild-type controls

			Lys (%)	*p* value	Hyl (%)	*p* value	GHL (%)	*p* value	GGHL (%)	*p* value
α1 K87	P3H3	WT	15.7 ± 13.3	9.78E-05	2.35 ± 0.24	1.01E-05	7.35 ± 1.08	1.03E-04	74.6 ± 12.3	4.49E-05
		Null	77.8 ± 3.06		10.3 ± 1.15		2.30 ± 0.28		9.65 ± 1.78	
	LH1	WT	8.28 ± 5.34	4.34E-08	2.06 ± 0.37	1.59E-04	6.83 ± 0.36	2.77E-08	82.8 ± 5.18	6.40E-08
		Null	98.9 ± 0.12		0.47 ± 0.78		0.20 ± 0.01		0.42 ± 0.03	
α2 K87	P3H3	WT	6.41 ± 3.13	2.95E-08	93.6 ± 3.13	2.95E-08	ND		ND	
		Null	73.0 ± 1.92		27.0 ± 1.92		ND		ND	
	LH1	WT	2.40 ± 0.28	3.33E-15	97.6 ± 0.28	3.33E-15	ND		ND	
		Null	98.0 ± 0.24		2.04 ± 0.24		ND		ND	
α1 K99	P3H3	WT	76.5 ± 2.31	3.83E-05	20.4 ± 1.43	1.27E-05	2.86 ± 0.91	0.00916	0.32 ± 0.11	0.08426
		Null	91.0 ± 1.40		7.68 ± 1.31		1.11 ± 0.12		0.19 ± 0.06	
	LH1	WT	78.4 ± 0.82	1.21E-08	17.5 ± 1.06	1.46E-07	3.54 ± 0.23	4.29E-07	0.62 ± 0.05	9.04E-06
		Null	96.5 ± 0.26		2.70 ± 0.13		0.64 ± 0.11		0.18 ± 0.03	
α1 K174	P3H3	WT	66.7 ± 4.73	3.38E-04	32.2 ± 4.34	2.82E-04	1.16 ± 0.44	0.00656	ND	
		Null	84.2 ± 0.84		15.5 ± 0.79		0.24 ± 0.10		ND	
	LH1	WT	63.1 ± 0.50	3.00E-11	34.4 ± 0.50	5.13E-11	1.88 ± 0.06	9.56E-09	0.53 ± 0.02	
		Null	92.1 ± 0.07		7.69 ± 0.11		0.24 ± 0.04		ND	
α2 K174	P3H3	WT	40.6 ± 13.1	2.16E-04	4.86 ± 1.24	0.48365	50.6 ± 10.6	9.59E-05	3.94 ± 1.76	
		Null	92.6 ± 1.27		5.40 ± 0.73		2.00 ± 1.21		ND	
	LH1	WT	22.7 ± 1.17	4.31E-11	7.89 ± 0.80	3.12E-04	60.0 ± 0.79	9.10E-12	9.47 ± 0.48	2.07E-08
		Null	92.9 ± 0.58		4.59 ± 0.39		2.35 ± 0.23		0.20 ± 0.02	
α1 K219	P3H3	WT	86.2 ± 1.55	0.00779	13.8 ± 1.55	0.00779	ND		ND	
		Null	90.0 ± 1.11		10.0 ± 1.11		ND		ND	
	LH1	WT	85.3 ± 0.48	1.67E-07	14.7 ± 0.48	1.67E-07	ND		ND	
		Null	95.1 ± 0.54		4.91 ± 0.54		ND		ND	
α2 K219	P3H3	WT	55.1 ± 7.05	0.00133	34.0 ± 4.12	0.0033	0.62 ± 0.21	0.00161	10.3 ± 2.77	5.15E-04
		Null	75.2 ± 0.91		23.9 ± 1.09		0.03 ± 0.04		0.88 ± 0.25	
	LH1	WT	50.3 ± 0.79	6.51E-10	34.7 ± 0.92	2.31E-08	0.91 ± 0.13	1.70E-05	13.9 ± 0.63	1.42E-08
		Null	85.4 ± 0.65		13.7 ± 0.64		0.08 ± 0.02		0.80 ± 0.09	
α1 K564	P3H3	WT	77.3 ± 1.20	0.7026	20.3 ± 1.06	0.33603	1.72 ± 0.13	0.07732	0.70 ± 0.12	0.13392
		Null	77.6 ± 0.79		19.7 ± 0.65		1.93 ± 0.14		0.85 ± 0.13	
	LH1	WT	78.9 ± 0.82	1.20E-07	18.4 ± 0.58	4.12E-08	2.00 ± 0.19	7.87E-06	0.69 ± 0.10	3.30E-04
		Null	92.4 ± 0.47		6.76 ± 0.35		0.55 ± 0.07		0.28 ± 0.05	
α2 K933	P3H3	WT	ND		93.1 ± 0.37	2.12E-09	6.86 ± 2.37		ND	
		Null	86.7 ± 1.56		13.3 ± 1.56		ND		ND	
	LH1	WT	ND		93.0 ± 1.23	7.36E-12	7.02 ± 1.23		ND	
		Null	97.9 ± 0.28		2.05 ± 0.28		ND		ND	


			Lys-Lys (%)	*p* value	Lys-Hyl (%)	*p* value	Hyl-Hyl (%)	*p* value
α1 K918/K930	P3H3	WT	1.57 ± 0.73	5.07E-07	8.11 ± 2.66	0.09689	90.3 ± 3.17	3.67E-07
		Null	88.9 ± 7.73		5.16 ± 1.41		5.99 ± 6.36	
	LH1	WT	0.29 ± 0.21	1.11E-16	4.00 ± 0.18	4.47E-08	95.7 ± 0.21	3.33E-16
		Null	99.0 ± 0.06		0.78 ± 0.07		0.22 ± 0.12	

GGHL, glucosylgalactosyl hydroxylysine; GHL, galactosyl hydroxylysine; Hyl, unmodified hydroxylysine; Hyl-Hyl, unmodified hydroxylysine and unmodified hydroxylysine; Lys, unmodified lysine; Lys-Hyl, unmodified lysine and unmodified hydroxylysine; Lys-Lys, unmodified lysine and unmodified lysine.

Values are given as means ± S.D. Biological replicates were n = 4 for all tissues and genotypes.

The lysine modification sites α1 K918 and K930 were included in the same peptide GD**K**GETGEQGDRGI**K**GER.

Lys + Hyl + GHL + GGHL = 100% and Lys-Lys + Lys-Hyl + Hyl-Hyl = 100%. Values of modified and unmodified hydroxylysine and unmodified lysine were obtained using mass spectrometry.

**Table 5:** Comparison of lysine posttranslational modifications at individual sites of tendon type I collagen from P3H3 and LH1 null mice relative to their wild-type controls

			Lys (%)	*p* value	Hyl (%)	*p* value	GHL (%)	*p* value	GGHL (%)	*p* value
α1 K87	P3H3	WT	0.56 ± 0.17	3.91E-10	96.8 ± 0.26	4.19E-10	1.70 ± 0.07	4.84E-07	0.93 ± 0.02	7.49E-07
		Null	49.1 ± 1.29		49.7 ± 1.25		0.77 ± 0.04		0.46 ± 0.04	
	LH1	WT	4.03 ± 1.86	5.84E-11	93.1 ± 1.85	6.77E-11	1.76 ± 0.02	1.70E-11	1.10 ± 0.02	1.12E-09
		Null	99.4 ± 0.13		0.45 ± 0.11		0.04 ± 0.01		0.12 ± 0.02	
α2 K87	P3H3	WT	0.35 ± 0.04	6.42E-12	99.7 ± 0.04	6.42E-12	ND		ND	
		Null	36.9 ± 0.49		63.1 ± 0.49		ND		ND	
	LH1	WT	0.30 ± 0.07	9.03E-18	99.7 ± 0.07	9.03E-18	ND		ND	
		Null	99.3 ± 0.12		0.66 ± 0.12		ND		ND	
α1 K99	P3H3	WT	76.8 ± 0.48	8.47E-09	18.6 ± 0.47	1.18E-08	1.47 ± 0.02	5.96E-10	0.10 ± 0.01	1.02E-04
		Null	92.8 ± 0.33		6.53 ± 0.33		0.60 ± 0.01		0.05 ± 0.01	
	LH1	WT	79.7 ± 0.56	7.94E-10	18.6 ± 0.53	1.05E-09	1.59 ± 0.08	2.31E-08	0.12 ± 0.01	
		Null	99.1 ± 0.18		0.80 ± 0.17		0.08 ± 0.07		ND	
α1 K174	P3H3	WT	60.5 ± 1.20	3.13E-05	38.8 ± 1.18	3.43E-05	0.71 ± 0.03	2.22E-04	ND	
		Null	67.7 ± 0.51		31.7 ± 0.54		0.53 ± 0.03		ND	
	LH1	WT	59.5 ± 1.31	1.53E-08	39.8 ± 1.31	1.81E-08	0.76 ± 0.05		ND	
		Null	86.8 ± 0.32		13.2 ± 0.32		ND		ND	
α2 K174	P3H3	WT	15.2 ± 0.26	2.95E-12	82.2 ± 0.17	2.90E-12	2.53 ± 0.10	8.17E-09	0.06 ± 0.01	7.01E-05
		Null	81.6 ± 0.74		18.2 ± 0.74		0.22 ± 0.03		0	
	LH1	WT	11.6 ± 0.57	7.91E-12	85.3 ± 0.40	6.56E-12	3.02 ± 0.21		0.05 ± 0.01	
		Null	92.1 ± 0.97		7.92 ± 0.97		ND		ND	
α1 K219	P3H3	WT	73.1 ± 1.10	0.6388	26.9 ± 1.10	0.6388	ND		ND	
		Null	72.7 ± 1.07		27.3 ± 1.07		ND		ND	
	LH1	WT	75.9 ± 1.16	2.90E-07	24.1 ± 1.16	2.90E-07	ND		ND	
		Null	92.7 ± 0.71		7.28 ± 0.71		ND		ND	
α2 K219	P3H3	WT	51.6 ± 0.51	4.67E-08	30.5 ± 0.54	0.00259	17.1 ± 0.16	1.02E-11	0.84 ± 0.02	5.20E-09
		Null	64.1 ± 0.54		32.5 ± 0.62		3.27 ± 0.12		0.14 ± 0.02	
	LH1	WT	48.3 ± 1.30	5.04E-08	31.4 ± 1.19	2.65E-06	19.2 ± 0.06	2.61E-14	1.09 ± 0.07	
		Null	87.1 ± 1.94		12.6 ± 1.86		0.38 ± 0.08		ND	
α1 K564	P3H3	WT	79.1 ± 1.09	0.01211	20.1 ± 1.05	0.01518	0.63 ± 0.04	0.00221	0.19 ± 0.01	1.12E-04
		Null	76.7 ± 0.81		22.3 ± 0.79		0.77 ± 0.03		0.27 ± 0.01	
	LH1	WT	80.1 ± 1.61	1.05E-06	18.8 ± 1.57	1.15E-06	0.66 ± 0.05	4.86E-07	0.22 ± 0.02	1.83E-04
		Null	96.5 ± 0.19		3.30 ± 0.19		0.06 ± 0.01		0.14 ± 0.01	
α2 K933	P3H3	WT	0.99 ± 0.12	2.22E-16	93.7 ± 1.15	9.40E-12	5.34 ± 1.20	2.84E-04	ND	
		Null	85.6 ± 0.16		13.6 ± 0.13		0.81 ± 0.08		ND	
	LH1	WT	0.52 ± 0.05	6.31E-18	91.6 ± 0.45	2.04E-14	7.92 ± 0.48		ND	
		Null	99.2 ± 0.12		0.75 ± 0.12		ND		ND	


			Lys-Lys (%)	*p* value	Lys-Hyl (%)	*p* value	Hyl-Hyl (%)	*p* value
α1 K918/K930	P3H3	WT	11.3 ± 0.29	1.14E-06	12.7 ± 0.58	0.13635	76.0 ± 0.75	5.17E-10
		Null	90.5 ± 8.07		7.43 ± 6.15		2.05 ± 1.94	
	LH1	WT	7.63 ± 0.41	3.32E-13	11.3 ± 0.41	3.26E-09	81.0 ± 0.75	3.06E-12
		Null	99.2 ± 0.63		0.30 ± 0.08		0.51 ± 0.60	

GGHL, glucosylgalactosyl hydroxylysine; GHL, galactosyl hydroxylysine; Hyl, unmodified hydroxylysine; Hyl-Hyl, unmodified hydroxylysine and unmodified hydroxylysine; Lys, unmodified lysine; Lys-Hyl, unmodified lysine and unmodified hydroxylysine; Lys-Lys, unmodified lysine and unmodified lysine.

Values are given as means ± S.D. Biological replicates were n = 4 for all tissues and genotypes.

The lysine modification sites α1 K918 and K930 were included in the same peptide GD**K**GETGEQGDRGI**K**GER.

Lys + Hyl + GHL + GGHL = 100% and Lys-Lys + Lys-Hyl + Hyl-Hyl = 100%. Values of modified and unmodified hydroxylysine and unmodified lysine were obtained using mass spectrometry.

### The absence of P3H3 and LH1 shows a slightly different molecular ensemble in lysyl hydroxylases and their associated proteins in the skin

**Figure 8:**
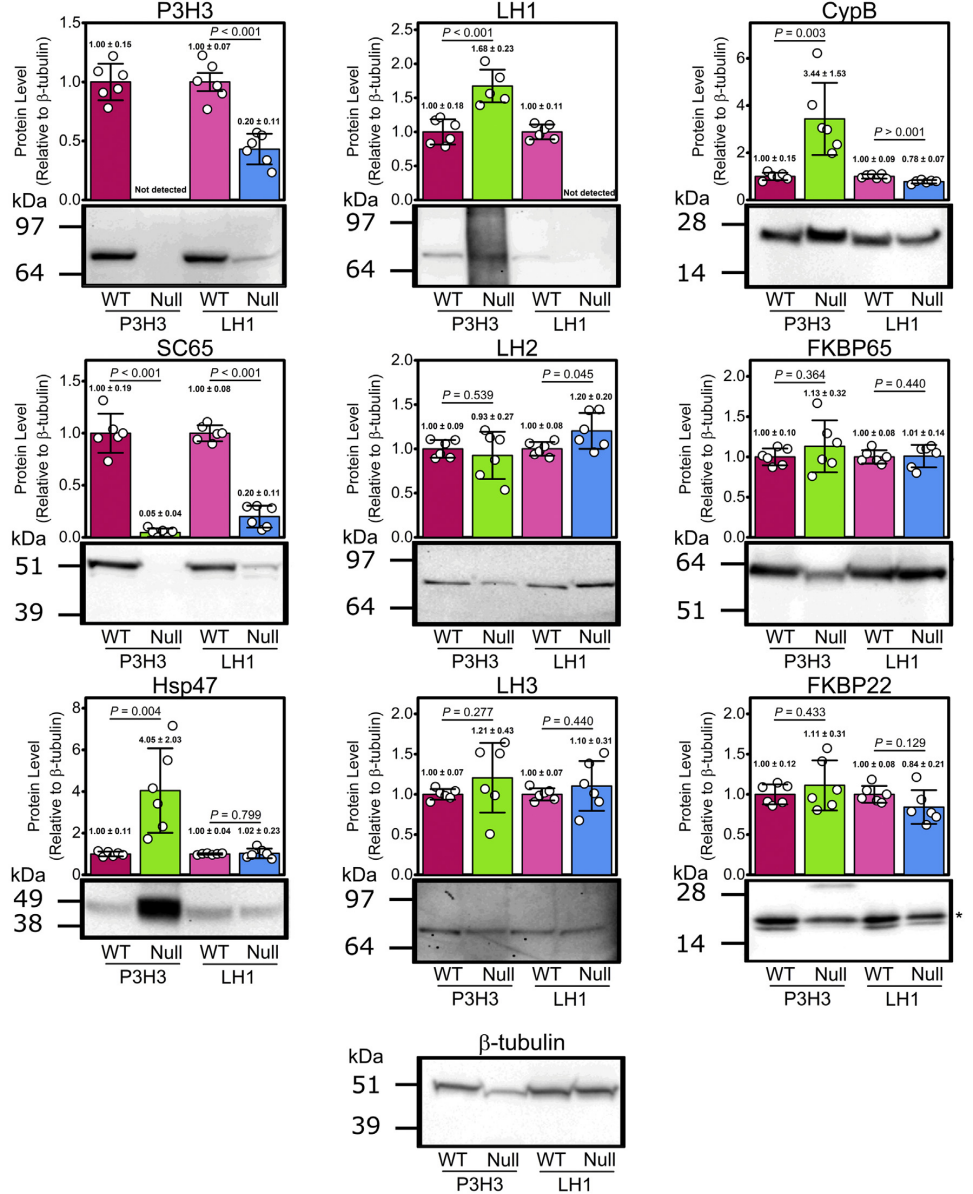
**Comparison of protein levels of skin LHs and their associated molecules in P3H3 null and LH1 null mice.** Semi-quantitative western blotting was performed to compare the protein levels in the molecular ensemble involved in lysyl hydroxylation using total soluble proteins extracted from the skin from P3H3 null mice and LH1 null mice and their WT controls. The protein signals were normalized to β-tubulin signals and each value was set to 1 for the WT controls. Data presented are means ± SD and individual data points represent independently prepared skin lysates (n = 6). Representative image of western blotting is shown under the bar graph. The whole western blotting images used for Figure 8 are shown in Fig. S9. *Asterisk* indicates N-glycosylated FKBP22 (63), which was used to this quantitative analysis.

To understand how type-dependent and site-differential effects occurred on lysyl hydroxylation in the collagen triple helix in the skin from P3H3 null and LH1 null mice, we quantified the protein level of P3H3, LH1, and CypB by western blots. In addition, the other LH1-associated molecule SC65 (38, 39), LH2 and proposed LH2-associated molecules FKBP65 and Hsp47 (50) and LH3 were also evaluated since all three isoforms have been suggested to hydroxylate the triple helical regions (15, 18, 25, 31, 32). Levels of LH2 and LH3 were not altered in P3H3 null and LH1 null mice compared with their WT controls (Fig. 8). Of the LH2-associated proteins, the FKBP65 protein level was the same in both null mice and Hsp47 was slightly increased in P3H3 null mice compared with their WT controls. We also checked FKBP22 because it belongs to the same EDS classification as LH1 (51, 52) but no difference in FKBP22 was observed between P3H3 null and LH1 null mice and their WT controls (Fig. 8). However, LH1 and its associated molecules showed intriguing changes. SC65 was markedly decreased in both null mice. Moreover, we found the opposite trend in other molecules when compared between P3H3 null and LH1 null mice. The protein level of LH1 and CypB was enriched in P3H3 null mice, while that of P3H3 and CypB was significantly reduced in LH1 null mice compared with their WT controls (Fig. 8). This could explain why the site preference effect and global reduction on lysyl hydroxylation of type I collagen were observed in P3H3 null and LH1 null mice, respectively. Unfortunately, we were unable to clarify how lysyl hydroxylation of type V collagen was diminished despite the abundance of LH1 and CypB proteins in P3H3 null mice.

### Skin analysis in P3H3 null mice

**Figure 9:**
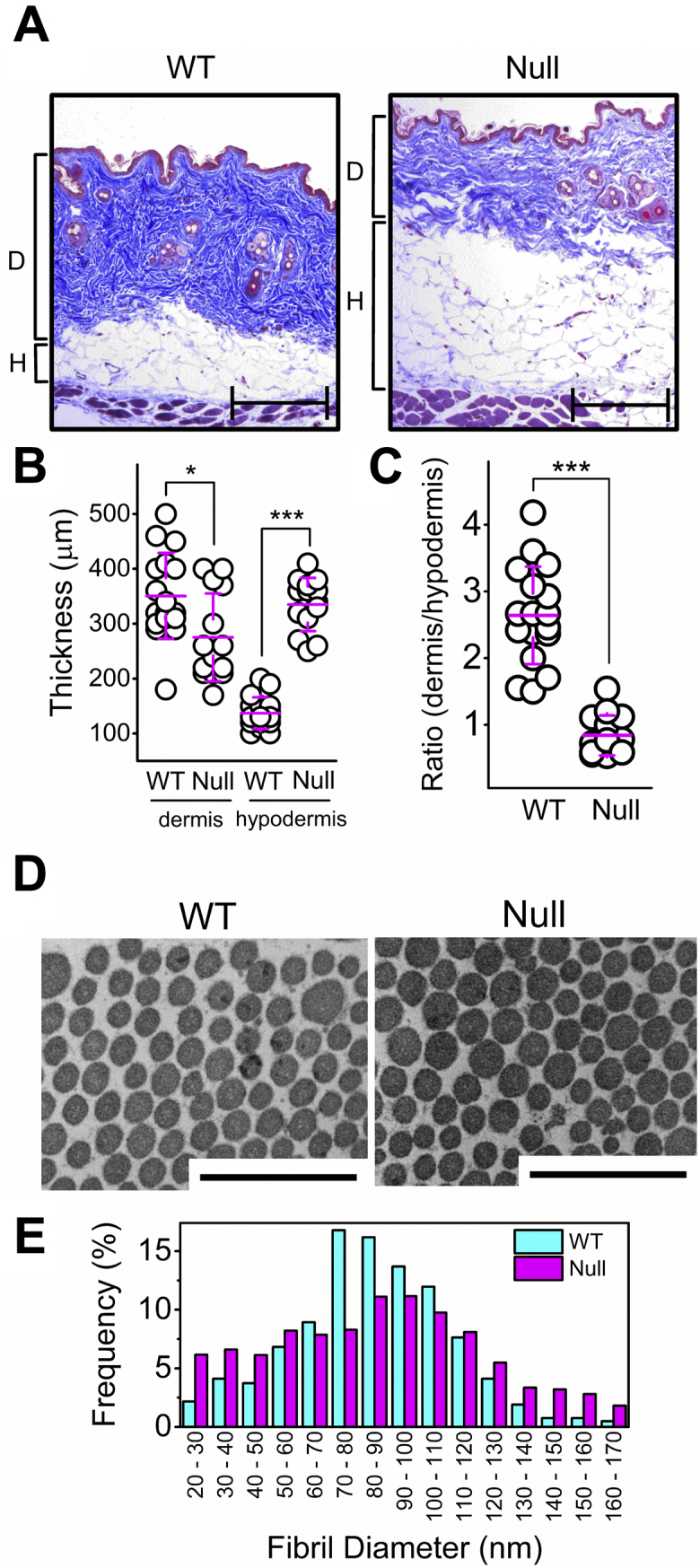
**Skin analysis of P3H3 null mice.***A*, Masson’s trichrome staining was performed using skin sections from 8-month-old P3H3 WT and null mice. D and H indicate dermis and hypodermis corresponding to the *blue rich* and the *white rich area*, respectively. Scale bars, 200 μm. *B*, the thickness of dermis and hypodermis was measured from the images of Masson’s trichrome stained skin sections. The averaged thicknesses of dermis and hypodermis are 350.6 ± 78.2 μm and 137.1 ± 28.7 μm for WT and 275.0 ± 80.1 μm and 335.7 ± 48.6 μm for P3H3 null mice. ∗ and ∗∗∗ indicate *p* < 0.05 (*p* = 0.013) and *p* < 0.0005 (*p* = 1.50 E^−14^), respectively. *C*, the ratio between dermis and hypodermis was calculated from Masson’s trichrome stained skin sections. The averaged ratio between dermis and hypodermis is 2.64 ± 0.73 for WT and 0.84 ± 0.30 for P3H3 null. ∗∗∗ indicates *p* < 0.0005 (*p* = 1.96 E^−9^). *D*, electron microscopy images displaying the skin collagen fibrils in 3-month-old P3H3 WT and null mice. Scale bar, 500 nm. *E*, the histogram represents the fibril diameter distribution in P3H3 WT (*cyan*) and null (*magenta*) mice. The averaged fibril diameters of P3H3 null and WT mice are 84.9 ± 35.6 nm and 85.1 ± 25.9 nm, respectively.

Given the differences in skin type V and type I collagen from P3H3 null mice, we performed skin analysis in P3H3 null mice. Consistent with our skin type V and I collagen analysis, and as reported previously (39), we found defects in the skin from P3H3 null mice. Masson’s Trichrome staining shows reduced collagen content, and the thickness and ratio of the dermis and hypodermis were altered (Fig. 9, *A*–*C*). Electron microscopy showed that the average diameter of collagen fibrils is similar between P3H3 null (84.9 ± 35.6 nm) and WT (85.1 ± 25.9 nm) mice (Fig. 9*D*). However, the distribution of fibril diameters was broader in P3H3 null mice (Fig. 9*E*), and this was also reported in LH1 null skin (37). In summary, a precise number of PTMs formed in collagen chains in the rER is a fundamental factor in maintaining an appropriate ultrastructure in collagen-rich tissues.

## Discussion

To clarify how LH1 functionally cooperates with the LH1-associated protein P3H3, we conducted quantitative analyses and directly compared the level of PTMs between WT, P3H3 null, and LH1 null mouse tissues. As a result, we discovered novel collagen type-dependent and site-differential effects of LH1 and P3H3 for precise lysyl hydroxylation in the collagen triple helix in the rER. We showed that type I collagen (without telopeptide regions) from the tendon and skin from LH1 and P3H3 null mice was differentially modified between the null mice and tissues (Fig. 6). We also found a decrease in the total amount of hydroxylysine in type I collagen from P3H3 null mouse tissues (Fig. 4). However, this decrease was smaller than in LH1 null mouse tissues (Fig. 4). In contrast to LH1 null and P3H3 null, CypB null mice have been reported to show more tissue-dependent alteration in the total amount of hydroxylysine: hydroxylysine was reduced in type I collagen from the tendon and skin, while an increase was observed in the bone (28, 29, 30). Taken together, lysyl hydroxylation in the triple helical domain of type I collagen is likely to be sensitive to the disruption of LH1 as well as LH1-associated proteins P3H3 and CypB. As shown in Figure 7 there are two regions in the triple helical domain of type I collagen: the cross-link-related edge regions that demonstrate the inextricable connection between LH1, P3H3, and CypB, and the central helical region diversely affected by the absence of LH1, P3H3, and CypB. Since some ECM proteins (integrins, decorin, and SPARC) potentially interact with type I collagen near the central K174 site (53) and this site is particularly affected in the absence of P3H3, LH1, or CypB (Fig. 7), α2(I) K174 could perform a biologically important function.

**Figure 10:**
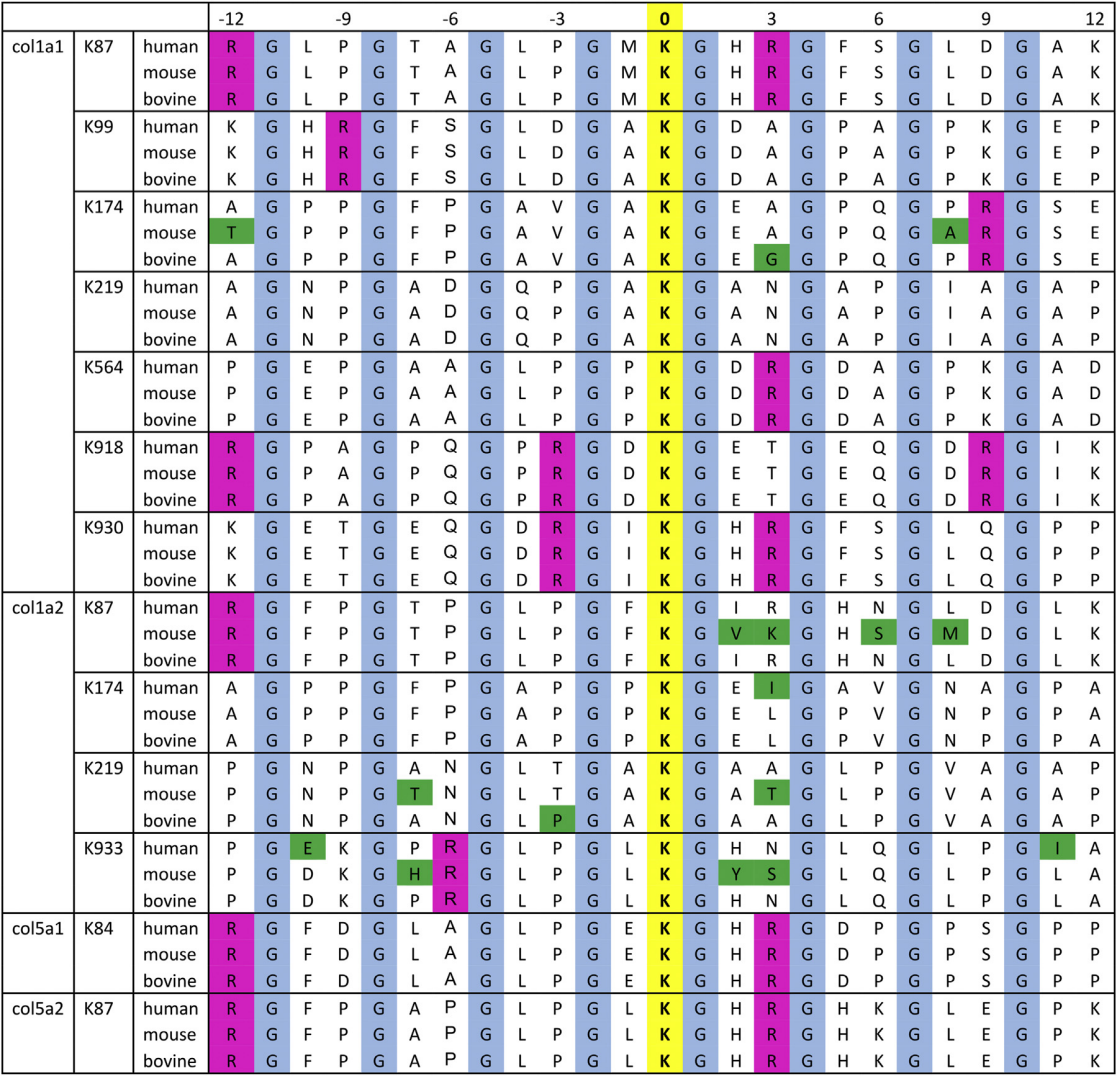
**Sequence alignment surrounding lysyl hydroxylation sites in type I and type V collagen in human, mouse, and bovine.** The sequences are aligned ±12 residues from the lysine residue, which is modified to hydroxylysine and highlighted by *yellow* with *bold*. Glycine residues in GXY repeats and the residues not conserved between human, mouse, and bovine are highlighted by *cyan* and *green*, respectively. Arginine residues in RGXY sequences are also highlighted by *magenta* because these residues are critical for Hsp47 to bind to collagen triple helices (64). Here we note that a previous report showed the difference in PTMs at α1(V) K87 (39). However, the actual residue 87 is arginine instead of lysine, as we showed above. This is also confirmed by the database below. Uniprot entry numbers are as follows: human COL1A1 (P02452), mouse COL1A1 (P11087), bovine COL1A1 (P02453), human COL1A2 (P08123), mouse COL1A2 (Q01149), bovine COL1A2 (P02465), human COL5A1 (P20908), mouse COL5A1 (O88207), human COL5A2 (P05997), and mouse COL5A2 (Q3U962). NCBI accession numbers are as follows: bovine COL5A1 (XP_024855494) and bovine COL5A2 (XP_024835542).

We also looked for specific binding or enhancer sequence motifs in type I and V collagen close to the lysyl hydroxylation sites based on our results (Fig. 10). As suggested in a previous report (28), a KGH sequence occurs at or near the cross-link formation sites and could provide preferential interaction sites for the CypB-involved SC65/P3H3 ER complex to facilitate LH1 activity. This hypothesis is possible but cannot alone explain the reduction of α2(I) K87 and K174 since the KGH sequence only exists near α2(I) K87 in mouse and does not exist near α2(I) K174 (Figs. 7 and 10). In type V collagen, the α1(V) K84 and α2(V) K87 are involved in cross-link formation (45), and these sites are also in a KGH sequence (Fig. 10). However, the effects on hydroxylation of these sites are not consistent between the P3H3 and LH1 null (Fig. 3*C*). Rather than the presence of specific binding or enhancer sequences, we envision that a three-dimensionally and temporally precise molecular interplay between LH1, P3H3, and CypB is required, particularly around the cross-link formation sites according to Figure 7, because lysyl hydroxylation should occur before the formation of a triple helix. This biologically intriguing event could be explained as not merely chaperone effects and/or complex formation, but as a dynamic and responsive regulation such as in the case of biomolecular condensates and protein phase separation (54, 55). Collectively, we would like to term this precise mechanism as a “local molecular ensemble.” Further studies are required to elucidate how LH1 and LH1-associated proteins properly distinguish and gather to form a local molecular ensemble at individual lysyl hydroxylation sites at a molecular level.

Previous studies of LH1 null mice and EDS type VIA patients suggested that tissue-specific and collagen–type-specific lysyl hydroxylation could exist since different magnitudes of reduction in lysyl hydroxylation between tissues were found (33, 34, 35, 36). Potential explanations were suggested such as the complexity of different collagen types between tissues, a distribution in the expression and/or protein levels of LH isoenzymes or compensation by the two other LH isoenzymes, which could hydroxylate peptides containing the sequences of hydroxylation sites of the triple helices of type I and type IV collagen (9, 18, 56). In the skin from both P3H3 null and LH1 null mice, we could not find any change in LH2 and LH3 proteins (Fig. 8). However, LH1 and its associated molecules were clearly varied in their protein levels depending on the absence of P3H3 and LH1 (Fig. 8). This could be interpreted in the global and site preference effect on lysyl hydroxylation of type I collagen in P3H3 null and LH1 null mice. Here, we propose an additional explanation that the combination in the expression and/or protein levels between LH1 and LH1-associated proteins also plays a vital role in providing precise PTMs in tissues. Surprisingly, P3H3 null mice showed an apparent reduction of lysyl hydroxylation in type V collagen from the skin, while there was no significant difference in type V collagen hydroxylation from LH1 null mice (Fig. 2*B*). Similar observations have been made before: the results from the lung and kidney of LH3 mutant mice demonstrated that the amount of hydroxylysine was not changed in type I collagen-rich fractions but was reduced by 30% in type IV and type V collagen-rich fractions (35). Together with our results, this strongly suggests that LH1 and LH3 have substrate specificity for at least type I collagen and type V collagen, respectively. Additionally, given that the LH3 mutant mice only showed a decrease of 30% in lysyl hydroxylation of type IV and type V collagen-rich fractions and that type V collagen from P3H3 null mice showed a reduction of hydroxylysine content, we speculate that either P3H3 supports hydroxylation of type V collagen by LH2 or that P3H3 may itself have lysyl hydroxylase activity and would be a LH-like enzyme (LH4) in the rER. Although P3H3 belongs to the prolyl 3-hydroxylase family, it has not been assigned a substrate or enzyme activity. Thus, direct prolyl and lysyl hydroxylase activity assays are needed to determine if P3H3 acts as a prolyl and/or lysyl hydroxylase. However, attempts to produce necessary quantities of recombinant P3H3 protein have not been successful, partly due to solubility issues (57).

In conclusion, P3H3 and LH1 both play critical roles in the hydroxylation of lysine residues at cross-link formation sites in type I collagen, whereas they likely have distinct mechanisms to modify other sites in type I collagen and to recognize different collagen types such as type V collagen in the rER. Indeed, it is still unclear what the most important factors are to obtain the precise PTM patterns in collagens: is it the formation of specific protein–protein interactions or the level of expression that leads to the existence of active complexes? Our findings offer new directions for the understanding of lysyl hydroxylation in various tissues and different collagens and provide a new interpretation of previous conclusions in LH1 null mice, LH3 null mice, and EDS VI patients.

## Experimental procedures

### Ethics statement

The protocols for the P3H3 null and corresponding WT mice were approved by the Oregon Health & Science Institutional Animal Care and Use Committee (Permit Number: ISO1999). Tissue isolation from the LH1 null and corresponding WT mice was performed under an animal experiment license number ESAVI/8179/04.10.07/2017 approved by the Animal Experiment Board of Finland, following the regulations of the EU Directive 86/609/EEC, the European Convention ETS123, and the national legislation of Finland. The LH1 null mice were housed in the University of Oulu Laboratory Animal Center, and the recommendations given by the Federation of European Laboratory Animal Science Associations and the Finnish and EU legislations concerning laboratory animal experiments and handling were followed.

### P3H3 null mice

P3H3 null mice were purchased from Ozgene. Directed knockouts were created in which exon 1 of the mouse *Leprel2* (also called *P3H3*) gene (UniProt entry number: Q8CG70), encoding P3H3, was deleted. The procedure is briefly described as follows. The PGK-neo selection cassette flanked by FRT sites was generated by PCR from C57BL/6J genomic DNA and inserted downstream of the exon 1, which was flanked by loxP sites. The targeted locus was eliminated using an FLP recombinase and a Cre recombinase. ES cell clones were confirmed by southern hybridization. P3H3 inactivation was verified in mice by DNA preparation from tissues and PCR with primer sets (Fwd: 5’-CTTACCCACACTAGACCCATGTGTC-3’ and Rev: 5’-GTTGCATTCATTAGCCTAGACCCGCTA-3’). The sizes of the PCR products for the WT and null alleles were approximately 1000 and 200 pb, respectively (Fig. 1 and Fig. S1).

### LH1 null mice

Generation of LH1 (*Plod1*) knockout mice using in-frame insertion of the lacZ-neo cassette into exon 2 has been described earlier (37). The LH1 mouse line Plod1(tm1Soin) (European Mouse Mutant Archive ID: EM:08327) was fully backcrossed to the C57BL/6JOlaHsd strain, but at the time of this work the C57BL/6JOlaHsd strain was changed to the C57BL/6N strain in the University of Oulu Animal Facility. Therefore, the background of the mice used in this study was C57BL/6JOlaHsd crossed with C57BL/6N one time. Genotyping was done by PCR (37), and tissues were harvested from 2.5-month-old mice for collagen analysis.

### Tissue and specimen preparation

Detailed information of the harvested tissue and specimen for each experiment is listed in Table S1

### Western blotting

A kidney was extracted from WT, heterozygous, and null P3H3 mouse and homogenized using T-PER (Thermo Fisher Scientific) containing protease inhibitors at 4 °C. After centrifugation, soluble proteins in the extract were mixed with NuPAGE LDS sample buffer with reducing agents. The protein solutions were separated by Bolt 4 to 12% Bis-Tris Plus (Thermo Fisher Scientific) gel electrophoresis and electrotransferred onto PVDF membranes. The membranes were blocked in PBS solution containing 5% (w/v) skim milk for antibody staining. All proteins were detected by alkaline phosphatase developed with 5-bromo-4-chloro-3-indolyl phosphate and Nitro blue tetrazolium. Rabbit polyclonal antibody against P3H3 (*LEPREL2*) and rabbit polyclonal antibody against GAPDH were purchased from Proteintech (16023-1-AP) and Sigma-Aldrich (PLA0125), respectively. Alkaline phosphatase-conjugated anti-rabbit IgG (A9919: Sigma-Aldrich) was used as a secondary antibody. Western blotting was performed three times using three independently prepared kidneys.

### X-ray scan

X-ray scanning was performed at least three times on independently prepared adult mice using a Faxitron cabinet instrument (model #43855B, Hewlett Packard). Voltages and exposure times were optimized for best-resolution images.

### Collagen extraction from tissues

Tendon, skin, and bone were taken from adult mice (Table S1). All procedures were performed at 4 °C. Tendon and skin were incubated in excess volume of 0.1 M acetic acid with shaking for several hours. Pepsin was added to a final concentration of 0.25 mg/ml, and tissues were digested overnight. Ground bone in liquid nitrogen was incubated in 1.0 M acetic acid containing 0.05 M EDTA. Pepsin digestion was performed after EDTA decalcification for 3 days. The pepsin-treated solutions were centrifuged to remove insoluble material, NaCl was added to a final concentration of 0.7 M to precipitate collagens, and the solution was incubated overnight. Precipitates were collected by centrifugation at 13,000 rpm for 15 min and resuspended in 0.1 M acetic acid. This solution contained an enriched type I collagen from the tendon and bone and was dialyzed against 0.1 M acetic acid to remove remaining NaCl. For the skin, the solution was dialyzed in an excess volume of 0.1 M Tris/HCl containing 1.0 M NaCl, pH 7.8, and NaCl was then added to a final concentration of 1.8 M to remove type III collagen. This solution was centrifuged at 13,000 rpm for 30 min, and additional NaCl was added to a final concentration of 2.4 M to the supernatant. After incubating overnight, the solution was centrifuged at 13,000 rpm for 30 min. The pellets containing skin type I collagen were resuspended in 0.1 M acetic acid and dialyzed against 0.1 M acetic acid to remove remaining NaCl. Skin type V collagen was extracted using the supernatant of 0.7 M NaCl precipitation, and additional NaCl was added to a final concentration of 4.0 M. After incubating overnight, the solution was ultracentrifuged at 30,000 rpm for 30 min. The pellets containing skin type V collagen were resuspended in 0.1 M acetic acid and dialyzed against 0.1 M acetic acid to remove remaining NaCl.

### Circular dichroism

CD spectra were recorded on an AVIV 202 spectropolarimeter (AVIV Biomedical, Inc) using a Peltier thermostat controlled cell holder and a 1-mm path length rectangular quartz cell (Starna Cells Inc). The temperature scanning curves were monitored at 221 nm with 10 °C/h scan rate. All curves shown were the average of at least three independent measurements using independently prepared type V and I collagen samples from tissues

### Amino acid analysis

Acid hydrolysis was performed in 6 x 50-mm Pyrex culture tubes placed in Pico Tag reaction vessels fitted with a sealable cap (Eldex Laboratories, Inc). Samples were placed in culture tubes, dried in a SpeedVac (GMI, Inc), and then placed into a reaction vessel that contained 250 ml of 6 M HCl (Pierce) containing 2% phenol (Sigma-Aldrich). The vessel was then purged with argon gas and evacuated using the automated evacuation workstation Eldex hydrolysis/derivatization workstation (Eldex Laboratories, Inc). Closing the valve on the Pico Tag cap maintained the vacuum during hydrolysis at 105 °C for 24 h. The hydrolyzed samples were then dried in a Savant SpeedVac. The dried samples were dissolved in 100 ml of 0.02 M HCl containing an internal standard (100 μM norvaline; Sigma). The analysis was performed by ion-exchange chromatography with postcolumn ninhydrin derivatization and visible detection (440 nm/570 nm) with a Hitachi L-8800A amino acid analyzer (Hitachi High Technologies America, Inc) running the EZChrom Elite software (Scientific Software, Inc). Three technical replicates were performed in each analysis.

### Glycosylation analysis

Glycosylation of hydroxylysine was estimated by LC-MS after alkaline hydrolysis, as described previously (58). In brief, type I and type V collagen samples were subjected to alkaline hydrolysis (2 N NaOH, 110 °C for 20 h under N_2_) after adding stable isotope-labeled collagen as an internal standard. The alkaline hydrolysates were neutralized with 30% acetic acid and then desalted using a mixed-mode cation-exchange sorbent (Oasis MCX; Waters). Unmodified and glycosylated hydroxylysines were quantitated by LC-MS in multiple reaction monitoring mode using a 3200 QTRAP hybrid triple quadrupole/linear ion trap mass spectrometer (AB Sciex) coupled to an Agilent 1200 Series HPLC system (Agilent Technologies) with a ZIC-HILIC column (3.5 μm particle size, L × I.D. 150 mm × 2.1 mm; Merck Millipore). Chromatographic separation was performed at a flow rate of 300 μl/min with a binary gradient as follows: 90% solvent B (100% acetonitrile) for 3 min, linear gradient of 10 to 95% solvent A (0.1% acetic acid/5 mM ammonium acetate) for 10 min, 95% solvent A for 4 min, and 90% solvent B for 3 min.

### Site-specific characterization of lysine PTMs

Site occupancy of lysine PTMs was estimated by LC-MS after protease digestion, as described previously (28). In brief, type I collagen solution samples were digested with trypsin (Promega) or with recombinant collagenase from *Grimontia hollisae* (Nippi) and pepsin (Sigma-Aldrich) after heat denaturation at 60 °C for 30 min. On the other hand, type V collagen was digested with trypsin not only in solution but also in SDS-PAGE gel. The samples were separated by SDS-PAGE, and the regions containing α1(V) and α2(V) were subjected to in-gel digestion with trypsin. The protease digests of type I and type V collagen were analyzed by LC-MS on a maXis II quadrupole time-of-flight mass spectrometer (Bruker Daltonics) coupled to a Shimadzu Prominence UFLC-XR system (Shimadzu). Peptides were separated on an Ascentis Express C18 HPLC Column (5 μm particle size, L × I.D. 150 mm × 2.1 mm; Supelco) at a flow rate of 500 μl/min with a binary gradient as follows: 100% solvent A (0.1% formic acid) for 2.5 min, linear gradient of 2 to 50% solvent B (100% acetonitrile) for 12.5 min, 90% solvent B for 2.5 min, and 100% solvent A for 2.5 min. Peptides were ionized and detected in positive ion mode with data acquisition using otofControl version 4.0 (Bruker Daltonics). The MS scan (type I and type V collagen samples) and MS/MS acquisition (type V collagen samples) were performed over the *m/z* ranges of 50 to 2500 with a frequency of 2 Hz (peptide quantification) or 5 Hz (peptide identification). The acquired MS/MS data were converted to.mgf files for database search against the UniProtKB/Swiss-Prot database (release 2018_05) for *Mus musculus* species (16,970 protein entries) using ProteinPilot software 4.0 (AB Sciex) with the Paragon algorithm (59). The MS data sets for identification of lysine PTM sites in type V collagen have been deposited to the repository (60, 61), and the more detailed information is in the Data Availability section below. Search parameters included digestion by trypsin, biological modifications ID focus, and 95% protein confidence threshold. Search criteria of PTMs were optimized for collagen analysis as described previously (62). Site occupancy of each modification site was calculated using the peak area ratio of monoisotopic extracted ion chromatograms of peptides containing the respective molecular species as previously reported for type I collagen (28). α1(I) K918/K930 and other sites were analyzed using the collagenase/pepsin digests and the trypsin digests, respectively.

### Protein level analyses in skin

All procedures for sample preparation were performed at 4 °C. Skin was taken from adult mice (Table S1) and proteins were extracted using RIPA buffer (Thermo Fisher Scientific) containing Halt Protease Inhibitor Cocktail, EDTA-Free (Thermo Fisher Scientific). After centrifugation, soluble proteins in the extract were mixed with SDS sample buffer with reducing agents. These protein solutions were separated on Bolt 4 to 12% Bis-Tris Plus Gels (Thermo Fisher Scientific), then transferred to PVDF membranes, and western blots were performed using antibodies and gel running buffers listed in Table S3. Blots were developed with HRP enhanced SuperSignal West Pico Chemiluminescent Substrate (Thermo Fisher Scientific) and detected by ChemiDoc MP imaging system (BioRad) using the software Image Lab version 4.0.1 (BioRad). The intensities of protein signals were measured by Image J.

### Masson’s trichrome staining

OHSU histology core facility performed sectioning and staining of the skin samples. Ventral skin tissues (n = 3) were fixed in 4% paraformaldehyde for 24 h at 4 °C. The fixed tissues were dehydrated using an ethanol gradient, cleared in xylene, and embedded in paraffin wax, after which 5 μm sections were cut using a microtome. The tissue sections were stained with Masson’s trichrome for collagen fiber analysis. Regions of interest (n = 2–4) were selected per image (n = 5), and the thickness of dermis and hypodermis was measured. The thickness was normalized by each scale bar.

### Electron microscopy analysis of skin

The three independently prepared skin samples from P3H3 null and WT mice were fixed in 1.5% glutaraldehyde/1.5% paraformaldehyde (Electron Microscopy Sciences) in Dulbecco’s serum-free media (SFM) containing 0.05% tannic acid. The samples were rinsed in SFM, postfixed in 1% OsO_4_, then dehydrated in a graded series of ethanol to 100%, rinsed in propylene oxide, and infiltrated in Spurr’s epoxy. Samples were polymerized at 70 °C for 18 h. Ultrathin sections (∼80 nm) were cut on a Leica EM UC7 ultramicrotome and mounted on formvar-coated, copper palladium 1 x 2 mm slot grids. Sections were stained in saturated uranyl acetate followed by lead citrate and photographed using an AMT 2K x 2K side entry camera (AMT) mounted on a FEI G2 transmission electron microscope operated at 120 kV, and at least four images were taken per sample.

### Fibril diameter measurement

The number and cross-sectional area of fibrils were measured using the Fiji software (ImageJ). Regions of interest were selected from the images of WT (n = 13) and P3H3 null (n = 17) skin, and fibril areas were measured by counting the pixels per fibril, which accounts for μm^2^. Spatial calibration for diameter measurements was applied against each scale bar. The numbers were counted for 1845 and 3616 fibrils of P3H3 WT and null samples, respectively.

### Statistical analyses

For comparisons between two groups, one-way ANOVA was performed to determine whether differences between groups are significant using ORIGIN Pro ver. 9.1 (OriginLab Corp). The *p* value of less than 0.05 was considered statistically significant.

## Data availability

The MS data sets for identification of lysine PTM sites in type V collagen have been deposited to the ProteomeXchange consortium *via* the jPOST partner repository with the data set identifier PXD019748 (https://repository.jpostdb.org/entry/JPST000744) (60, 61). Identification of lysine PTM sites in type I collagen was performed previously (28, 29). All other data are contained within the article and supporting information. The all source data are available from the corresponding author upon reasonable request.

## Supporting information

This article contains supporting information.

## Conflict of interest

The authors declare that they have no competing interests related to this work.
