# Supplementary material for: Type I and type V procollagen triple helix uses different subsets of the molecular ensemble for lysine posttranslational modifications in the rER
Source: J Biol Chem. 2021 Feb 23;296:100453. doi: 10.1016/j.jbc.2021.100453 (PMC7988497; doi:10.1016/j.jbc.2021.100453)
Supplement: Supplemental Figures S1–S9 and Tables S1–S3 [file mmc1.pdf]

Supporting Information for

Yoshihiro Ishikawa[1,2,3], Yuki Taga[4], Keith Zientek[2, #a], Nobuyo Mizuno[2, #b¶], Antti M. Salo[5], Olesya Semenova[2], Sara F. Tufa[2], Douglas R. Keene[2], Paul Holden[2], Kazunori Mizuno[4], Douglas B. Gould[3,6] Johanna Myllyharju[5] and Hans Peter Bächinger[1]

From [1] Department of Biochemistry and Molecular Biology, Oregon Health & Science University, Portland, Oregon, USA, [2] Shriners Hospital for Children, Research Department, Portland, Oregon, USA, [3] Department of Ophthalmology, University of California San Francisco, School of Medicine, California, USA, [4] Nippi Research Institute of Biomatrix, Ibaraki, Japan, [5] Oulu Center for Cell-Matrix Research, Biocenter Oulu and Faculty of Biochemistry and Molecular Medicine, University of Oulu, Oulu, Finland, [6] Department of Anatomy, University of California, San Francisco, School of Medicine, CA USA.

Running title: Two distinct roles of LH1and P3H3 in lysine hydroxylation

Present address #; [a] Proteomics core facility, Oregon Health & Science University, Portland, Oregon, USA, [b] Vaccine and Gene Therapy Institute, Oregon Health and Science University, Portland, Oregon, USA.

* To whom correspondence should be addressed: Yoshihiro Ishikawa, Ph.D., Department of Ophthalmology, University of California, San Francisco, School of Medicine, California, USA; Tel: (503) 866-5940, E-mail: yoshi.molecularensemble@gmail.com and yoshihiro.ishikawa@ucsf.edu, ORCID-ID: https://orcid.org/0000-0003-2013-0518

Table S1: Detailed information of harvested tissue and specimen

Experiment	Mouse line	Age	Related Figure and Table
Western blotting	P3H3	3-month-old	Figures 1C and S1B
X-ray	P3H3	4-month-old	Figure 1E
Collagen extraction # and Protein level analysis	P3H3	2-5-month-old	Figures 2, 3, 4, 5, 6, 8, S2, S3, S4, S5, S6, S7, S8 and S9 / Table 1, 2, 3, 4, 5 and S1
	LH1	2.5-month-old	
Skin staining	P3H3	8-month-old	Figures 9A, 9B and 9C
EM	P3H3	3-month-old	Figure 9D
Fibril diameter	P3H3		Figure 9E

#: Extracted collagens were used for SDS-PAGE analysis, amino acid analysis, circular dichroism, glycosylation analysis and site-specific characterization of lysine PTMs.

Table S2: Comparison of overall proline and lysine post-translational modifications between P3H3 WT and LH1 WT mice in type I collagen between tissues and type V collagen in the skin.

			3Hyp (%)	P value	4Hyp (%)	P value	Pro (%)	P value	Hyl (%)	P value	Lys (%)	P value
Col5	skin	P3H3 WT (5)	1.14 ± 1.1	0.91743	44.8± 4.5	0.03363	54.1 ± 5.0	0.0508	60.4 ± 4.7	0.86983	39.6 ± 4.7	0.8186
		LH1 WT (3)	1.07 ± 0.20		52.5 ± 2.1		46.5 ± 2.3		60.0 ± 2.6		40.5 ± 2.6	
Col1	skin	P3H3 WT (4)	0.17 ± 0.17	0.04954	47.1 ± 3.3	0.06191	52.7 ± 3.5	0.05605	16.9 ± 4.4	0.63679	83.1 ± 2.6	0.60438
		LH1 WT (4)	0.57 ± 0.27		51.1 ± 1.1		48.3 ± 1.3		18.2 ± 2.4		81.7 ± 2.4	
	tendon	P3H3 WT (6)	0.95 ± 0.57	0.41272	44.5 ± 2.7	0.04573	54.6 ± 3.4	0.06719	19.7 ± 2.6	0.18342	80.3 ± 2.5	0.17535
		LH1 WT (6)	1.17 ± 0.26		47.2 ± 0.9		51.7 ± 1.1		18.1 ± 0.6		81.9 ± 0.6	
	bone	P3H3 WT (5)	1.46 ± 0.84	0.20661	48.1 ± 1.72	0.98643	50.5 ± 2.1	0.60563	22.0 ± 3.3	0.09326	78.0 ± 3.3	0.12138
		LH1 WT (5)	0.85 ± 0.53		48.0 ± 1.2		51.1 ± 1.5		18.3 ± 2.8		81.3 ± 2.6	

Values are given as means ± S.D. The number in the bracket next to the genotypes indicates biological replicates.

3Hyp + 4Hyp + Pro = 100 % and Lys + Hyl = 100 %. Values of amino acids were obtained using amino acid analysis.

Note: 3Hyp; 3-hydroxyproline, 4Hyp; 4-hydroxyproline, Pro; unmodified proline, Lys; unmodified lysine, Hyl; hydroxylysine

Table S3: Antibodies and gel running buffers used for Western blot analyses.

Name	company	Product number	dilution ratio	Gel running buffer
anti-LH1 (PLOD1) Mouse monoclonal	Santa Cruz Biotechnology	sc-271640	1:250	MOPS
anti-P3H3 (LEPREL2) Rabbit Polyclonal	proteintech	16023-1-AP	1:500	MOPS
anti-SC65 (LEPREL4) Rabbit Polyclonal	proteintech	15288-1-AP	1:500	MOPS
anti-CypB (PPIB) Rabbit Polyclonal	Sigma-Aldrich	SAB4200201	1:1000	MES
anti-LH2 (PLOD2) Rabbit Polyclonal	proteintech	21214-1-AP	1:500	MOPS
anti-FKBP65 (FKBP10) Rabbit Polyclonal	proteintech	12172-1-AP	1:1000	MOPS
anti-Hsp47 (SERPINH1) Rabbit Polyclonal	proteintech	10875-1-AP	1:500	MES
anti-LH3 (PLOD3) Rabbit Polyclonal	proteintech	11027-1-AP	1:500	MOPS
anti-FKBP22 (FKBP14) Rabbit Polyclonal	proteintech	15884-1-AP	1:500	MES
anti-β-Tubulin III Rabbit Polyclonal	Sigma-Aldrich	T2200	1:500	MOPS
Secondary antibody				
HRP-conjugated anti-mouse IgG	Dako	P0447	1:10,000	
HRP-conjugated anti-rabbit IgG	GE Healthcare	NA934	1:30,000	

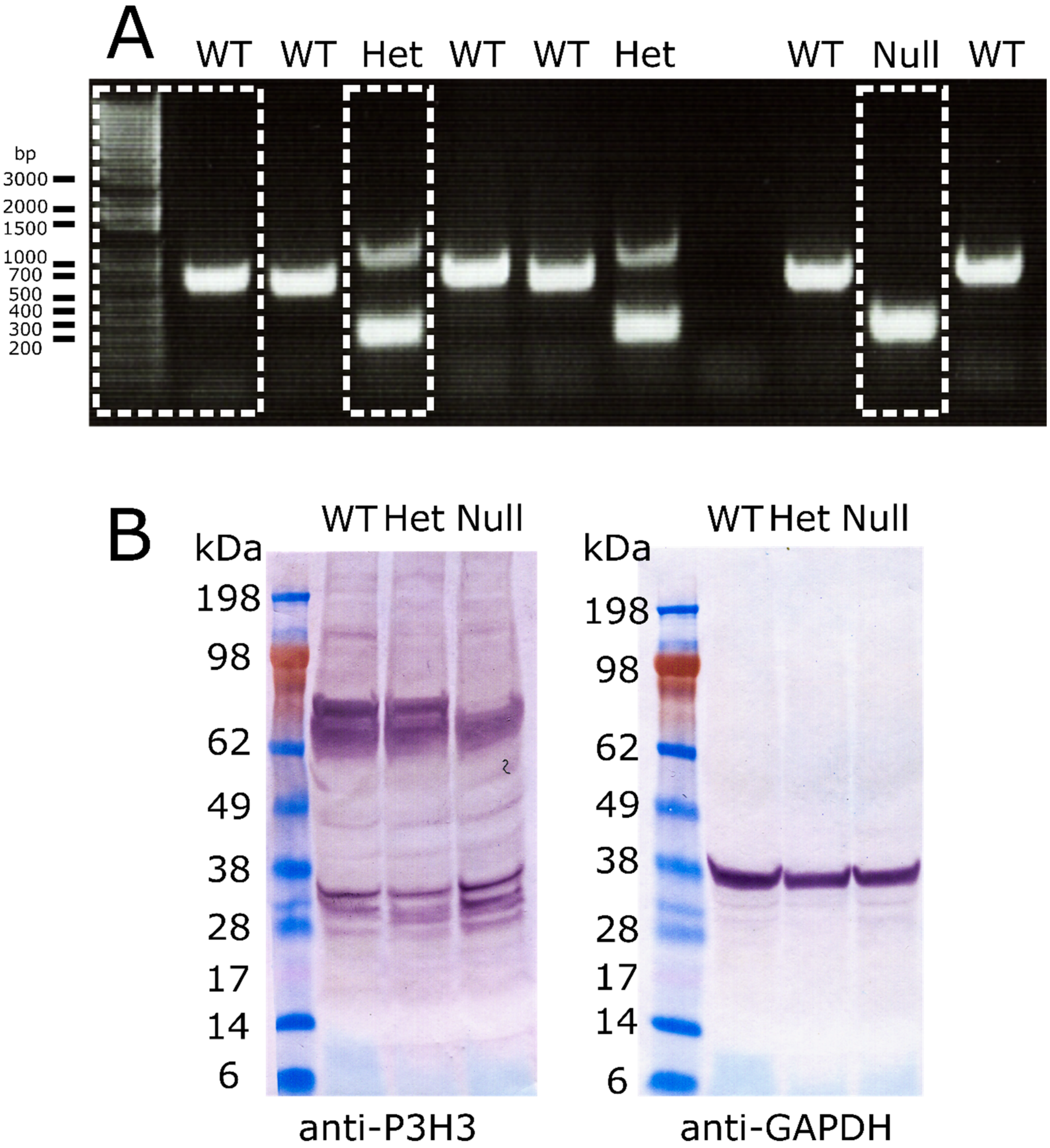

Figure S1. Original agarose gel and Western blotting images used in Figure 1.

(A) Original agarose gel image of PCR genotyping of P3H3 null, heterozygote and WT mice. The lanes circled by white dots are used in Figure 1B. (B) Original image of a Western blot of whole mouse kidney lysates analyzed with anti-P3H3 (left) anti-GAPDH (right), respectively.

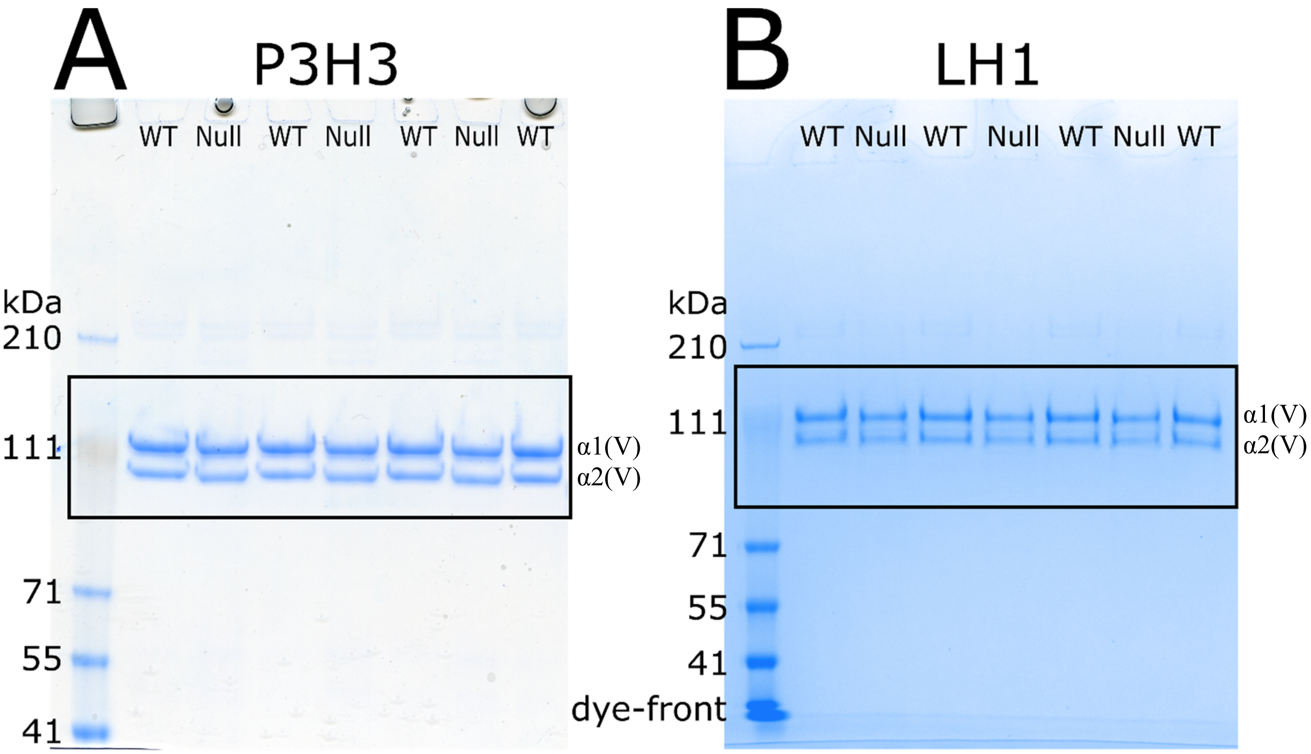

Figure S2. SDS-PAGE analysis of purified pepsin treated skin type V collagen of P3H3 and LH1 null mice.

SDS-PAGE analysis of purified pepsin treated skin type V collagen of P3H3 and LH1 null mice and their WT controls. The purified skin type V collagen was run on a NuPAGE 3 – 8 % Tris-Acetate gel in the presence of a reducing agent and stained with GelCode Blue Stain Reagent. Each sample in the SDS-PAGE gel represents a biological replicate, i.e. an independently prepared collagen sample from the tissue. Panel (A) and (B) represent the comparison between P3H3 WT and null, and between LH1 WT and null mice, respectively. The area circled by black line is used in Figure 2A.

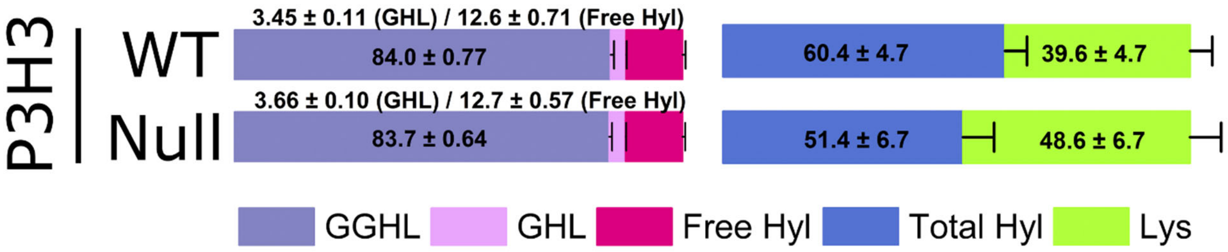

	GGHL (%)	P-value	GHL (%)	P-value	Free Hyl (%)	P-value	Total Hyl (%)	Lys (%)
WT	84.0 ± 0.77	0.587	3.45 ± 0.11	0.068	12.6 ± 0.71	0.817	60.4 ± 4.7	39.6 ± 4.7
Null	83.7 ± 0.64		3.66 ± 0.10		12.7 ± 0.57		51.4 ± 6.7	48.6 ± 6.7

Figure S3. Occupancy (%) of glycosylation in total hydroxylysine residues in skin type V collagen from P3H3 null mice.

The occupancy of *O*-glycosylation attached to hydroxylysine (GGHL + GHL + free Hyl = 100) in skin type V collagen of WT and P3H3 null mice is demonstrated in the left bar graph generated by values from the table underneath. The right bar graph shows the ratio of total PTMs in lysine (Lys + Hyl = 100) and is the same as in Figure 2B. Values of *O*-glycosylation and unmodified hydroxylysine after alkaline hydrolysis were obtained by LC-MS. The number of biological replicates is n = 3 for both genotypes. The numbers in the graphs indicate the mean ± S.D. P values obtained by statistical analyses are shown in the table underneath. The values of total hydroxylysine and lysine were from Table 1. [GGHL (gray); glucosylgalactosyl hydroxylysine, GHL (light pink); galactosyl hydroxylysine, Free Hyl (dark pink); unmodified hydroxylysine, Total Hyl (blue); total hydroxylysine, Lys (green); unmodified lysine].

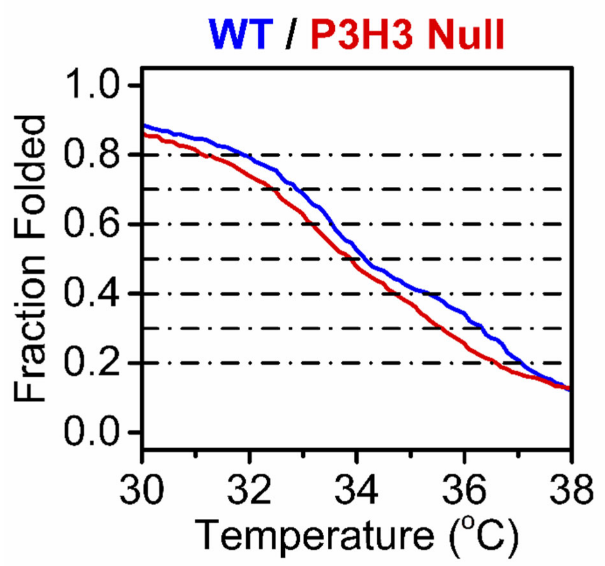

Fraction Folded	Temperature (°C)		P value
	**WT**	**P3H3 Null**	
0.8	**31.9 ± 2.3**	**31.4 ± 2.1**	0.77
0.7	**33.3 ± 2.2**	**32.6 ± 2.1**	0.70
0.6	**34.1 ± 2.0**	**33.4 ± 1.8**	0.68
0.5	**34.6 ± 1.9**	**34.1 ± 1.7**	0.74
0.4	**35.2 ± 1.8**	**34.7 ± 1.6**	0.71
0.3	**35.9 ± 2.0**	**35.4 ± 1.9**	0.76
0.2	**36.6 ± 2.3**	**36.3 ± 2.2**	0.87

Figure S4. The melting temperature of each fraction folded in skin type V collagen from P3H3 null mice.

The thermal stability of skin type V collagen from P3H3 WT (blue) and null (red) mice was monitored by CD at 221 nm in 0.05 M acetic acid between 30 and 38 °C (left panel). The whole graph used for this analysis is shown in Figure 2C. The table on the right presents the melting temperature of each fraction folded in the CD curve of P3H3 WT and null skin type V collagen. The number of biological replicates is n = 3 for both WT and P3H3 null. The numbers in the graphs indicate the mean ± S.D. P values obtained by statistical analyses are also shown.

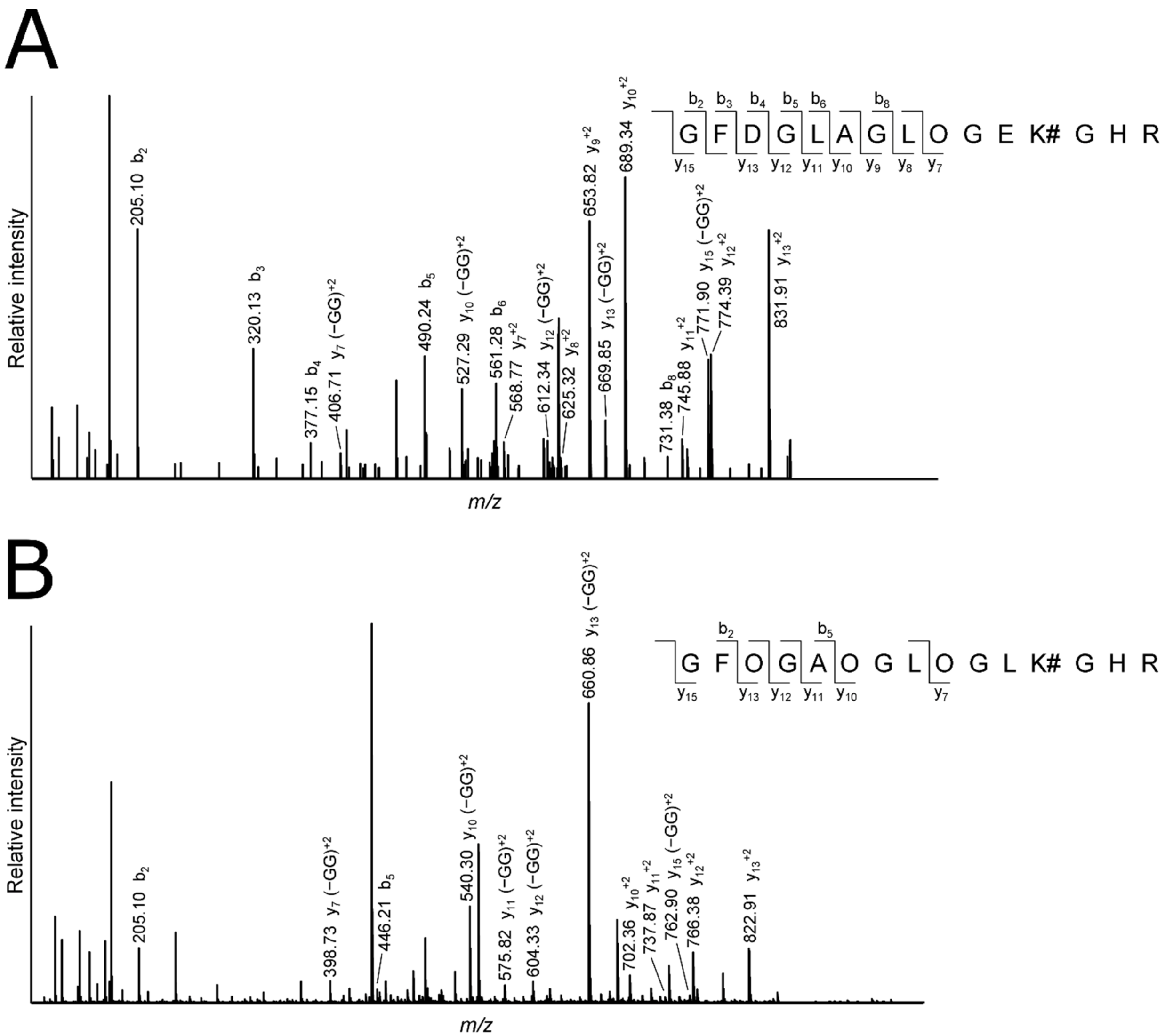

Figure S5. MS/MS spectra of GGHL-containing tryptic peptides of type V collagen.

(A) α1(V) [73–87] GFDGLAGLOGEK#GHR (z = 3, m/z 622.9722; O indicates Hyp, and K# indicates GGHL). (B) α2(V) [76–90] GFOGAOGLOGLK#GHR (z = 3, m/z 616.9731). −GG represents deglycosylated fragment ions.

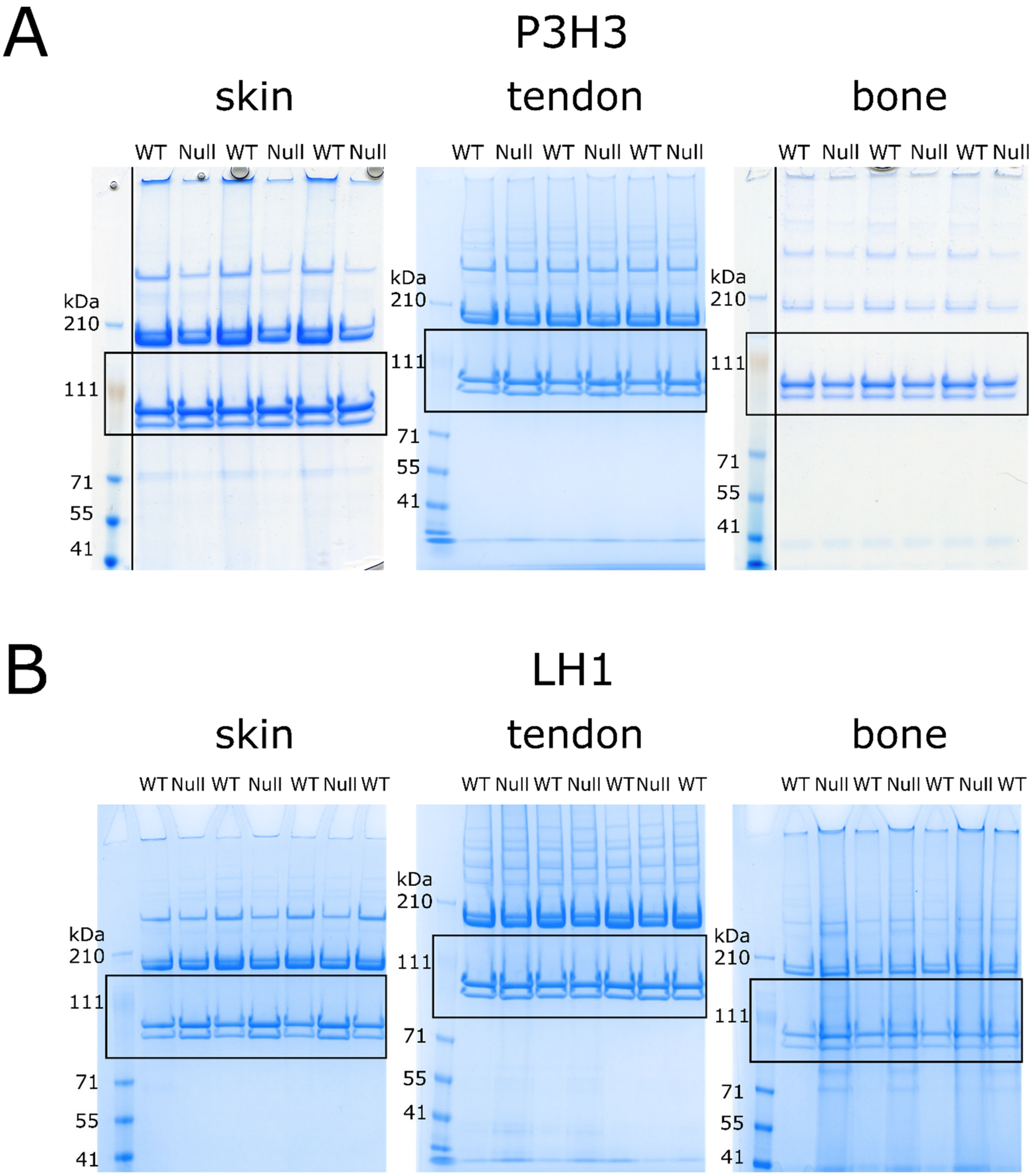

Figure S6. SDS-PAGE analysis of purified pepsin treated type I collagen of P3H3 and LH1 null mice. SDS-PAGE analysis of purified pepsin treated type I collagen from skin, tendon and bone of P3H3 and LH1 null mice and their WT controls. The purified type I collagen was run on a NuPAGE 3 – 8 % Tris-Acetate gel in the presence of a reducing agent and stained with GelCode Blue Stain Reagent. Each sample in the SDS-PAGE gel represents a biological replicate, i.e. an independently prepared collagen sample from

the tissue. Panel (A) and (B) represent the comparison between P3H3 WT and null, and between LH1 WT and null mice, respectively. The black vertical lines in panel (A) denotes irrelevant lanes that were eliminated from the original image. The area circled by black line is used in Figure 4A.

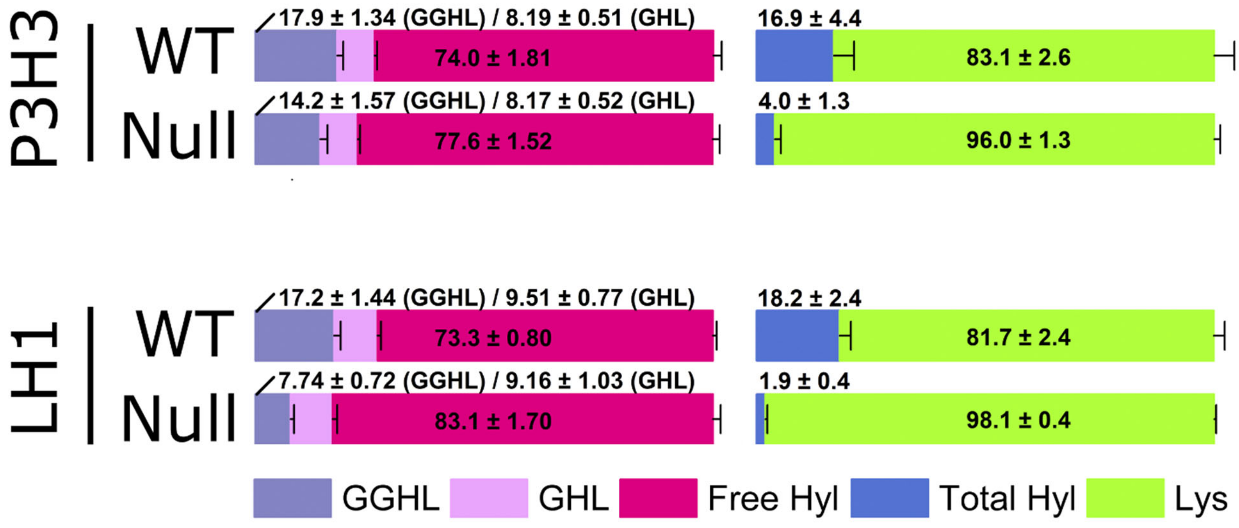

P3H3							
WT	Total Hyl (%):16.9 ± 4.4						Lys (%): 83.1 ± 2.6
WT	17.9 ± 1.34	P-value 0.012	8.19 ± 0.51	P-value 0.953	74.0 ±1.81	P-value 0.021	
	GGHL (%)		GHL (%)		Free Hyl (%)		
Null	14.2 ± 1.57		8.17 ± 0.52		77.6 ± 1.52		
Null	Total Hyl (%):4.0 ± 1.3						Lys (%): 96.0 ± 1.3

LH1							
WT	Total Hyl (%):18.2 ± 2.4						Lys (%): 81.7 ± 2.4
WT	17.2 ± 1.44	P-value 2.21E-5	9.51± 0.77	P-value 0.602	73.3 ± 0.80	P-value 4.36E-5	
	GGHL (%)		GHL (%)		Free Hyl (%)		
Null	7.74 ± 0.72		9.16 ± 1.03		83.1 ±1.70		
Null	Total Hyl (%): 1.9 ± 0.4						Lys (%): 98.1 ± 0.4

Figure S7. Occupancy (%) of glycosylation in total hydroxylysine residues in skin type I collagen from P3H3 and LH1 null mice.

The occupancy of *O*-glycosylation attached to hydroxylysine (GGHL + GHL + Free Hyl = 100) in skin type 1 collagen of P3H3 null and LH1 null mice and their WT controls is demonstrated in the left bar graph which is generated by values from the table underneath. The right bar graph shows the ratio of total PTMs in lysine (Lys + Hyl = 100) and is the same as in Figure 4B. Values of *O*-glycosylation and unmodified hydroxylysine after alkaline hydrolysis were obtained by LC-MS., The number of biological replicates was

n = 4 for all genotypes. The numbers in the graphs indicate the mean ± S.D. P values obtained by statistical analyses are shown in the table underneath. The values of total hydroxylysine and lysine in the right bar graph are from Table 1. [GGHL (gray); glucosylgalactosyl hydroxylysine, GHL (light pink); galactosyl hydroxylysine, Free Hyl (dark pink); unmodified hydroxylysine, Total Hyl (blue); total hydroxylysine, Lys (green); unmodified lysine].

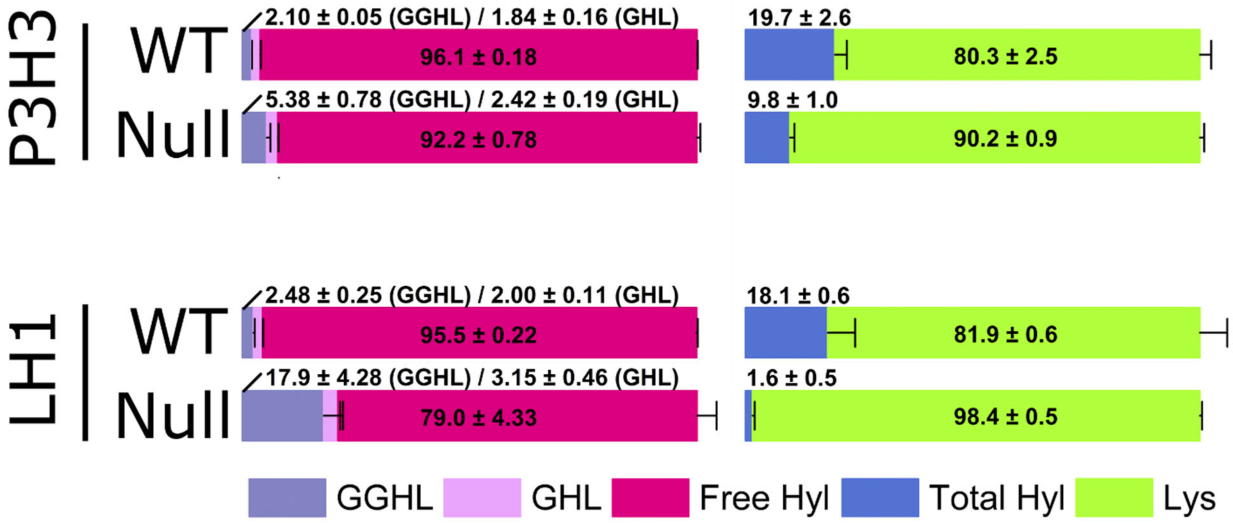

P3H3							
WT	Total Hyl (%):19.7 ± 2.6						Lys (%): 80.3 ± 2.5
	2.10 ± 0.05	P-value 1.50E-4	1.84 ± 0.16	P-value 0.003	96.1 ± 0.18	P-value 7.10E-5	
	GGHL (%)		GHL (%)		Free Hyl (%)		
Null	5.38 ± 0.78		2.42 ± 0.19		92.2 ± 0.78		
	Total Hyl (%): 9.8 ± 1.0						Lys (%): 90.2 ± 0.9

LH1							
WT	Total Hyl (%):18.1 ± 0.6						Lys (%): 81.9 ± 0.6
	2.48 ± 0.25	P-value 3.69E-4	2.00 ± 0.11	P-value 0.003	95.5 ± 0.22	P-value 2.64E-4	
	GGHL (%)		GHL (%)		Free Hyl (%)		
Null	17.9 ± 4.28		3.15± 0.46		79.0 ± 4.33		
	Total Hyl (%): 1.6 ± 0.5						Lys (%): 98.4 ± 0.5

Figure S8. Occupancy (%) of glycosylation in total hydroxylysine residues in tendon type I collagen from P3H3 and LH1 null mice.

The occupancy of *O*-glycosylation attached to hydroxylysine (GGHL + GHL + Free Hyl = 100) in tendon type 1 collagen of P3H3 null and LH1 null mice and their WT controls is demonstrated in the left bar graph which is generated by values from the table underneath. The right bar graph shows the ratio of total PTMs in lysine (Lys + Hyl = 100) and is the same as in Figure 4B. Values of *O*-glycosylation and unmodified hydroxylysine after alkaline hydrolysis were obtained by LC-MS. The number of biological replicates was

n = 4 for all genotypes. The numbers in the graphs indicate the mean ± S.D. P values obtained by statistical analyses are in the table underneath. The values of total hydroxylysine and lysine in the right bar graph are from Table 1. [GGHL (gray); glucosylgalactosyl hydroxylysine, GHL (light pink); galactosyl hydroxylysine, Free Hyl (dark pink); unmodified hydroxylysine, Total Hyl (blue); total hydroxylysine, Lys (green); unmodified lysine].

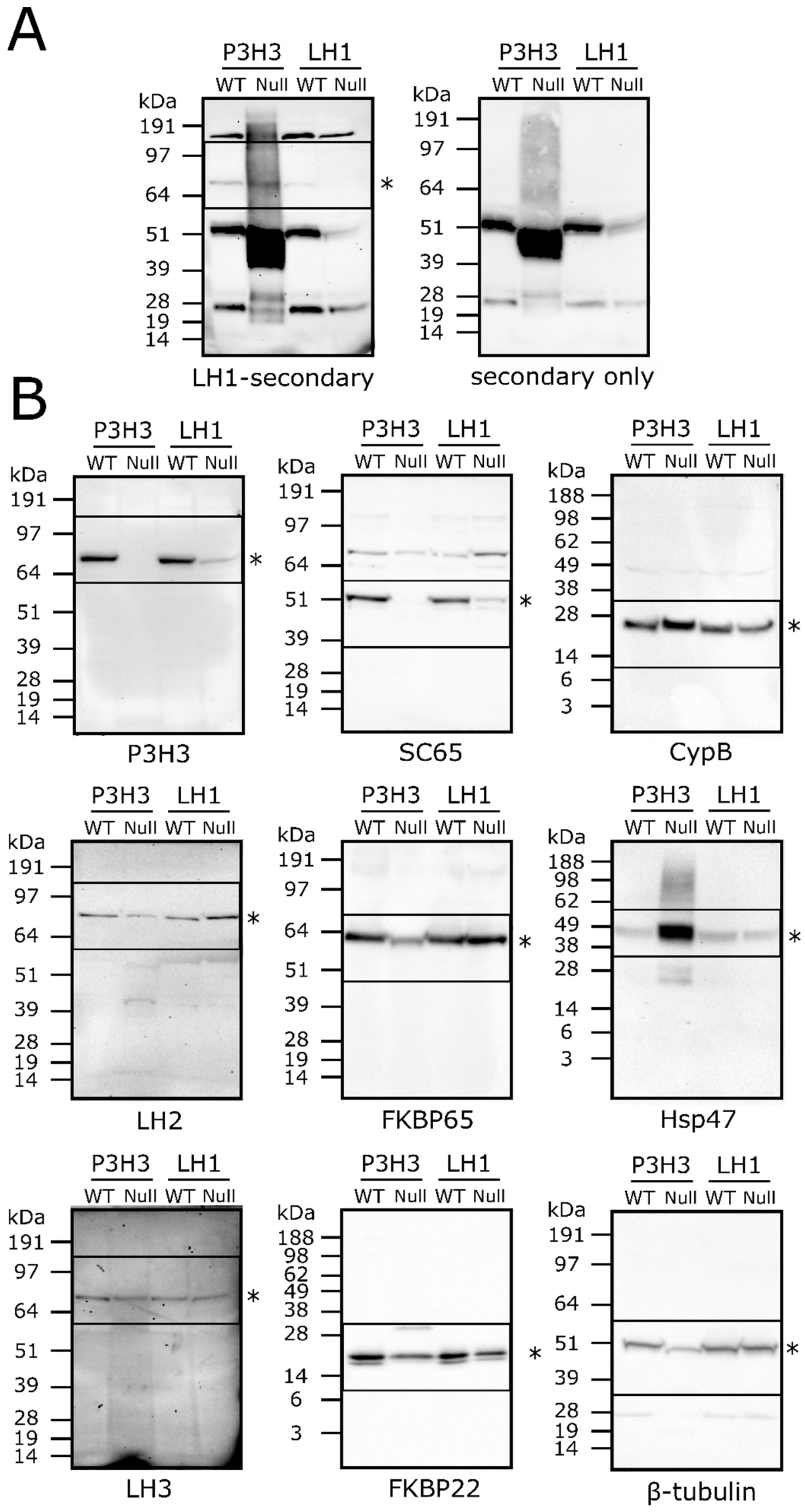
A
P3H3
LH1
WT
Null
kDa
191
97
64
51
39
28
19
14
*
LH1-secondary
secondary only
B
P3H3
SC65
CypB
LH2
FKBP65
Hsp47
LH3
FKBP22
β-tubulin
188
98
62
49
38
6
3

Figure S9. Western blots analysis of proteins in the molecular ensemble involved in lysyl hydroxylation using skin tissue extracts

The skin tissue extracted from WTs and P3H3 null mice and LH1 null mice were electrophoresed on a Bolt 4-12% Bis-Tris Plus Gel followed by transfer to PVDF and Western blotting. (A) and (B) shows the probing HRP-conjugated anti-mouse IgG and anti-rabbit IgG as a secondary antibody, respectively. To verify non-specific bands raised by the secondary antibody, the membrane probed with only secondary antibody is included in (A). Antibodies used to the blotting are listed in Supplemental Table S3. The asterisk indicates the migration corresponding to the molecular weight of the protein, which was used for further quantitative analysis.
